# Prevalence, risk factors and behavioural and emotional comorbidity of acute seizures in young Kenyan children: a population-based study

**DOI:** 10.1186/s12916-018-1021-y

**Published:** 2018-03-07

**Authors:** Symon M. Kariuki, Amina Abubakar, Martha Kombe, Michael Kazungu, Rachael Odhiambo, Alan Stein, Charles R. J. C. Newton

**Affiliations:** 1 KEMRI-Wellcome Trust Research Programme, PO Box 230, Kilifi, Kenya; 2 Department of Public Health, Pwani University, PO Box 195, Kilifi, Kenya; 3 Department of Psychiatry, University of Oxford, Oxford, OX3 7JX UK

**Keywords:** Acute seizures, Mental health problems, Children, Prevalence, Risk factors

## Abstract

**Background:**

Acute symptomatic seizures and febrile seizures are common in children admitted to hospitals in Africa and may be markers of brain dysfunction. They may be associated with behavioural and emotional problems, but there are no published community-based studies in Africa.

**Methods:**

We screened 7047 children aged 1–6 years (randomly sampled from 50,000 in the community) for seizures (using seven questions) and invited those who screened positive and a proportion of negatives for a clinical assessment. Risk factors were identified using a parental questionnaire. Behavioural and emotional problems were examined using the Child Behaviour Checklist (CBCL) in 3273 children randomly selected from 7047. Generalised linear models with appropriate link functions were used to determine risk factors and associations between behavioural or emotional problems and acute seizures. Sobel–Goodman mediation tests were used to investigate if the association between acute seizures and CBCL scores was mediated by co-diagnosis of epilepsy.

**Results:**

Acute seizures were identified in 429 (6.1%) preschool children: 3.2% (95% confidence interval CI: 2.9–3.5%) for symptomatic seizures, and 2.9% (95% CI: 2.6–3.3%) for febrile seizures. Risk factors for acute seizures included family history of febrile seizures (odds ratio OR = 3.19; 95% CI: 2.03–5.01) and previous hospitalisation (OR = 6.65; 95% CI: 4.60–9.63). Total CBCL problems occurred more frequently in children with acute seizures (27%; 95% CI: 21–34%) than for those without seizures (11%; 95% CI: 11–12%; chi-squared *p* ≤ 0.001). Acute seizures were associated with total CBCL problems (adjusted risk ratio (aRR) = 1.92; 95% CI: 1.34–2.77), externalising problems (aRR = 1.82; 95% CI: 1.21–2.75) and internalising problems (aRR = 1.57; 95% CI: 1.22–2.02), with the proportion of the comorbidity mediated by a co-diagnosis of epilepsy being small (15.3%; 95% CI: 4.5–34.9%). Risk factors for this comorbidity included family history of febrile seizures (risk ratio (RR) = 3.36; 95% CI: 1.34–8.41), repetitive acute seizures (β = 0.36; 95% CI: 0.15–0.57) and focal acute seizures (RR = 1.80; 95% CI: 1.05–3.08).

**Conclusions:**

Acute seizures are common in preschool children in this area and are associated with behavioural and emotional problems. Both conditions should be assessed and addressed in children.

**Electronic supplementary material:**

The online version of this article (10.1186/s12916-018-1021-y) contains supplementary material, which is available to authorized users.

## Background

Acute seizures are often provoked by extracranial febrile infections and metabolic abnormalities [1], and thus defined as febrile seizures and acute symptomatic seizures [2, 3]. Febrile seizures occur in 2–8% of children in the general population aged 6–72 months [3]. Acute symptomatic seizures occur in close temporal association with intracranial insults or metabolic perturbations [1], and the prognosis is thought to be poorer than for febrile seizures. Since it is difficult to differentiate between acute symptomatic and febrile seizures in Africa, it is proposed that seizures with fever in sub-Saharan Africa be referred to as acute seizures [2].

There are few epidemiological studies of acute seizures in Africa, where most estimates are from hospital studies, which can grossly underestimate burden [4]. The incidence of acute seizures in Kenyan children aged 0–13 years admitted to hospital is estimated to be between 425 and 650 per 100,000 per year [2, 5], which is higher than reports from high-income countries [6]. The prevalence of febrile seizures was estimated at 2.1% in a community-based study in Tanzania [7] and 8.4% in a hospital-based Togolese study [8]. The two studies did not investigate the risk factors for behavioural and emotional comorbidities of acute seizures, while some febrile seizures could have been acute symptomatic seizures since malaria was associated with over half of these seizures. Acute seizures in Africa increase the future risk for epilepsy [9] and mental health problems [10], so their early identification and prevention is of public health importance.

Behavioural and emotional problems were reported in 22% of German children with febrile seizures compared to 6% of the controls [11]. In sub-Saharan Africa, a greater proportion of acute seizures are complex (i.e. prolonged, repetitive or focal) (60–70%) [12, 13], which may increase the risk of behavioural and emotional problems in affected children [10]. Behavioural and emotional problems in children with seizures may be related to (i) the seizures, especially possible damage from complex seizures, (ii) a shared neurological damage that leads to both seizures and behavioural and emotional problems or (iii) an underlying genetic susceptibility that may interact with other environmental factors to lead to seizures, behavioural and emotional problems or both [10]. Behavioural and emotional problems occurred in approximately 10% of Kenyan children discharged from hospital with acute symptomatic seizures (a relative risk of 1.8 compared to controls) [10], but hospital estimates are biased towards more severe seizure disorders and it is unclear if behavioural and emotional problems were related to the seizures, malarial disease, shared genetic susceptibility or underlying neurological impairment. Since both acute seizures and behavioural and emotional problems are common on the Kenyan coast [5, 14], we examined their independent association, particularly in preschool children in whom mortality is high, and early initiation of interventions before they enrol school may be beneficial.

We conducted a population-based study to estimate the prevalence, determine risk factors and examine the behavioural and emotional comorbidity of acute seizures in preschool children residing in a rural area on the Kenyan coast. Furthermore, we investigated the risk factors associated with behavioural and emotional comorbidities of acute seizures.

## Methods

### Study area and population

This study was conducted in Kilifi, which is on the Kenyan coast, about 60 km north of Mombasa city [15]. The Kilifi Health and Demographic Surveillance System (KHDSS) has data on a population of about 270,000 residents, who are predominantly of the Mijikenda tribe. It is estimated that the population of children aged 1–6 years is about 50,000. The KHDSS has a northern and southern region divided by the Kilifi Creek, and it covers an area of about 891 km^2^. Kilifi County Hospital (KCH) is the only referral hospital in the area, and it draws admissions from Kilifi. There is an epilepsy and neurodevelopmental clinic, which provides treatment for seizures and counselling services for mental health problems [16].

### Sampling and sample size determination

We estimated that randomly selecting and screening 7587 children from 50,000 children aged 1–6 years within KHDSS in stage I would give the prevalence of seizure disorders (of at least 5%) with a precision of <1%. We had estimated that this survey would generate at least 250 children with acute seizures and an equal number of controls and that these numbers would be sufficient to measure an association between acute seizures and behavioural/emotional problems (with a risk ratio of ~2) with at least 80% at the 5% significance level. Those positive for seizures in stage I were invited for further clinical, neurological and anthropometrical evaluation in stage II, details for which are provided below. The 7587 children to participate in the study were randomly selected from a KHDSS database of 50,000 children living in the community aged 1 years using the RAND command of MySQL (Oracle Corp, USA). A parental risk factor questionnaire with items on sociodemographic, socioeconomic and medical history information was administered to all those with acute seizures and to a predetermined sample of those without seizures, who served as controls.

### Procedures

Seizures were screened in stage I by trained fieldworkers using a modified seven-question seizure questionnaire, which was piloted before the study (Additional file 1). The questions were based upon previous screening questionnaires for epilepsy studies [17] but were modified to capture seizures with fever occurring in the childhood period. Additional details on seizure onset, duration and localisation and family history were obtained in stage I, which could be used for those screening positive who failed to turn up for clinical evaluation in stage II (subsequently referred to as attrition). Electroencephalography (EEG) was performed on children with acute seizures who cooperated as previously described [18].

About half of the children screened for the presence or absence of a history of seizures in stage I were randomly selected for assessment for behavioural or emotional problems using the Child Behaviour Checklist (CBCL), which had been adapted and validated for use in this rural population and showed excellent psychometric properties in 301 children, some of whom were young [14, 19]. The strength of the CBCL is that it has questions that apply to young children such as ‘cries a lot’, ‘clings to adults or too dependent’, ‘doesn’t want to sleep alone’ etc. [19]. Administration of the CBCL (for behavioural and emotional assessment) was done on the same day of screening for seizures in stage I by the same fieldworker. As previously described [14], we used the CBCL scores, completed by the mother or primary caregiver, to form total CBCL problems, externalising problems, internalising problems, CBCL syndromes and Diagnostic and Statistical Manual of Mental Disorders IV (DSM-IV) oriented scales. A similar parental risk factor questionnaire was administered in stage I for those who screened negative for seizures and in stage II during clinical evaluation of those who screened positive in stage I.

### Clinical and neurological evaluation and anthropometric assessments

Children with a history of seizures and a random proportion (9%) of those without seizures were seen by a clinician in stage II for further clinical evaluation. The clinical evaluation in stage II was scheduled to happen within a week of screening in stage I. Those who failed to turn up were followed up and scheduled for evaluation as soon as possible. The clinician, who was blinded to seizures status in stage I, asked questions on the history of seizure disorders and determined whether the seizures were acute seizures or established epilepsy (according to the International League Against Epilepsy guidelines). The clinician obtained further information about onset of seizures, seizure type/frequency and duration. Definitions for seizure disorders and other conditions are in Additional file 2: Table S1. A clinician assessed cognitive impairments by observing how children performed on a number of items in a standardised developmental inventory [20]. A neurological examination was performed on all children invited for further review.

Causes of acute seizures were identified by asking the parent about the febrile illness that was associated with the seizures, and symptoms and signs at the time of the seizure (Additional file 2: Table S2). This followed the World Health Organization algorithms for Integrated Management of Child Infections (http://www.who.int/maternal_child_adolescent/topics/child/imci/en/), which were piloted among children admitted with malaria and correctly identified the majority of these children for treatment [21]. The community data on acute seizures were linked with hospital admissions to identify additional causes of acute seizures. Anthropometric measures of the child were also taken by neuropsychological assessors and included head circumference, mid-upper arm circumference, height and weight.

### Characterisation of acute seizures

Acute seizures (referred to, interchangeably, as provoked seizures) were febrile seizures if they were not associated with a diagnosis of malaria, meningitis or encephalopathy as identified with causes recorded in the hospital database or Integrated Management of Child Infections guidelines, while the remainder were presumed to be acute symptomatic seizures, since malaria is ubiquitous in this area. Convulsive seizures were classified into either focal or generalised, with detailed definitions provided in Additional file 2: Table S1.

### Statistical analysis

All analyses were performed using STATA (version 13, StataCorp, TX, USA). The prevalence of acute seizures was computed as a probability derived from the inverse link function of a logit model. Acute seizures are viewed as relatively benign in this community and are not treated at hospital, which may cause attrition between stage I and II. Attrition was addressed using multiple imputation by chained equations [22], implemented in STATA by constructing a model with age and seizure variables obtained in stage I. The assumption in the multiple imputation was that the missing data for acute seizure status in stage II occurred at random because failure to report for further clinical evaluation in the second stage also occurred at random. We included age and common phenotypes of seizures (e.g. prolonged seizures, focal seizures and repetitive seizures) to infer for missing acute seizure status in stage II because these variables were documented for all children in stage I and are often correlated with acute seizures [2].

The generalised linear models (GLMs) for risk factors for acute seizures had a logit link function to compute odds ratios (ORs), which closely approximate risk ratios when the outcome is relatively rare (<10%) [23]. The association of behavioural and emotional problems with acute seizures was determined by fitting a GLM of the binomial family (with a log link). Effect estimates for the association of behavioural and emotional problems with acute symptomatic seizures was hypothesised to be greater than that with febrile seizures, which was tested by computing ratios of the risk ratios (ROR) as previously described [24].

We investigated if the association between acute seizures and behavioural and emotional problems is mediated by a co-diagnosis of epilepsy using the Sobel–Goodman mediation tests [25]. The mediation analysis was necessary to understand which of the two seizure disorders (acute seizures or epilepsy) is associated with behavioural and emotional problems, since acute seizures can occur in children with a history of epilepsy [10] and both conditions can have poor behavioural outcomes. In those with acute seizures, risk factors for behavioural and emotional problems were determined with GLM models, with a binomial distribution and log link (when the outcome was binary), and with a Gaussian distribution and identity link (when the log-transformed CBCL scores were the outcome). In these models, McFadden’s pseudo *R*^2^ was used to test the variance of behavioural and emotional comorbidity of acute seizures explained by significant risk factors. Cohen’s *d* statistics was used to compare CBCL scores between groups. Risk factors reaching a *p* value ≤0.25 in the bivariate analysis were entered into a multivariable model. Pearson’s chi squared was used to compare discrete variables between groups, while Student’s *t*-test or the Mann–Whitney U test was used to compare continuous variables, such as age and behavioural/emotional scores. A *p* value of 0.05 or less was considered significant (unless a Bonferroni corrected cut-off is indicated), while an ROR > 1.0 signified a statistically significant difference between two ORs.

## Results

**Fig. 1:**
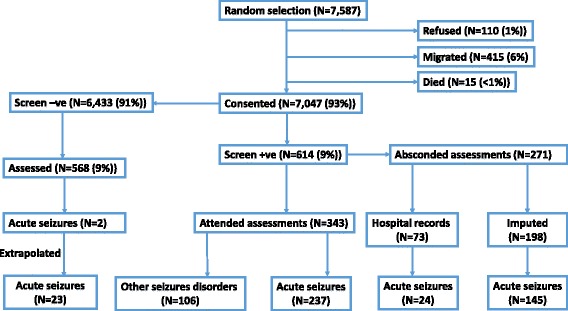
Flow chart for interviews of children and their guardians in the acute seizures study during stages I and II. Those who screened positive had their status determined through a clinical diagnosis, hospital records or multiple imputation. A random proportion of screen negatives was also seen by a clinician, and acute seizures extrapolated for all screen negatives. -ve negative, +ve positive

A history of seizures was taken for 7047/7587 (93%) children who had been randomly selected from the KHDSS database (Fig. 1). The median age of the 7047 children screened was 46 months (IQR 30–62), and 51% were males (Table 1). Socio-demographic, socioeconomic and medical characteristics for the children are shown in Table 1. The CBCL was administered to 3273/7047 (46%) of those randomly chosen.

**Table 1:** Socio-demographic and medical characteristics of study participants

Characteristics	Younger children ≤36 months (4539)	Older children >36 months (2508)	Total (*N* = 7047)	*p* value
Child’s age in months: median (IQR)	25 (19–31)	57 (47–68)	46 (30–62)	<0.001
Male	2315 (51%)	1279 (51%)	3594 (51%)	0.99
Mother’s age in years: median (IQR)	28 (23–34)	30 (25–36)	29 (25–35)	<0.001
Administered a risk factor questionnaire				
	*N* = 1122	*N* = 2085	*N* = 3207	
Socio-demographic information				
Mother’s marital status				0.09
Single	57 (5%)	95 (5%)	152 (5%)	
Separated/divorced	62 (6%)	139 (7%)	201 (6%)	
Widowed	13 (1%)	46 (2%)	59 (2%)	
Married	990 (88%)	1805 (87%)	2795 (87%)	
Mother’s religion				0.63
None	266 (24%)	532 (26%)	798 (25%)	
Traditional	64 (6%)	108 (5)	172 (5%)	
Islam	133 (9%)	254 (12%)	387 (12%)	
Christian	659 (59%)	1191 (57%)	1850 (58%)	
Mother’s education level				0.52
None	326/1120 (34%)	631/2080 (30%)	957/3200 (35%)	
Primary	707/1120 (63%)	1288/2080 (62%)	1995/3200 (62%)	
Secondary	67/1120 (6%)	135/2080 (6%)	202/3200 (6%)	
Tertiary	20/1120 (2%)	26/2080 (2%)	46/3200 (2%)	
Mother’s ethnicity				0.05
Ethnicity not Mijikenda	51 (5%)	115 (6%)	166 (5%)	
Other Mijikenda	77 (7%)	117 (6%)	194 (6%)	
Kauma	121 (11%)	190 (9%)	311 (10%)	
Chonyi	303 (27%)	643 (31%)	946 (30%)	
Giriama	570 (51%)	1018 (49%)	1588 (50%)	
Mother employed	761/1121 (68%)	1515/2080 (73%)	2276/3201 (71%)	0.003
Father employed	958/1031 (93%)	1786/1937 (92%)	2744/2968 (92%)	0.48
Number of children: median (IQR)	4 (2–6)	5 (3–6)	4 (3–6)	<0.001
Socioeconomic status and household information				
Water availability				0.49
Infrequent	205 (18%)	419 (20%)	624 (19%)	
Weekly	138 (12%)	242 (12%)	380 (12%)	
Daily	231 (21%)	447 (21%)	678 (21%)	
Always	548 (49%)	977 (47%)	1525 (48%)	
House status				
Dilapidated	76 (7%)	107 (5%)	183 (6%)	0.29
Major repairs needed	105 (9%)	195 (9%)	300 (9%)	
Incompletely built	29 (3%)	58 (3%)	87 (3%)	
Minor/no repairs needed	912 (81%)	1723 (83%)	2635 (82%)	
Toilet type				0.21
Bush/none	309/1121 (28%)	585/2082 (28%)	894/3203 (28%)	
Traditional pit	651/1121 (58%)	1161/2082 (56%)	1812/3203 (57%)	
Ventilated pit	105/1121 (9%)	195/2082 (9%)	300/3203 (9%)	
Flush	71 (5%)	75 (5%)	146 (5%)	
Livestock owned				0.21
None	507 (45%)	1004 (48%)	1511 (47%)	
<5	242 (22%)	406 (19%)	648 (20%)	
>5	373 (33%)	675 (32%)	1048 (33%)	
Pregnancy and birth history				
Pregnancy problems	88/1121 (8%)	140/2083 (8%)	228/3204 (7%)	0.24
Caffeine, smoking or alcohol in pregnancy	11/161 (7%)	26/335 (8%)	37/496 (7%)	0.71
Mother’s age at birth in years	18 (17–20)	18 (17–20)	18 (17–20)	0.02
Delivery at home	646 (58%)	1332 (64%)	1978 (62%)	<0.001
Premature birth	109/162 (67%)	214/334 (64%)	323/496 (65%)	0.48
Adverse perinatal events	42/1121 (4%)	48/2082 (2%)	90/3203 (2%)	0.02
Medical history				
Family history of seizures	127 (11%)	254 (12%)	381 (12%)	0.07
Family history of febrile seizures	102 (9%)	218 (10%)	320 (10%)	0.22
Previous hospitalisation	103/1119 (10%)	288/2076 (14%)	391/3195 (12%)	<0.001
Head injury	12/1122 (1%)	54/2083 (3%)	66/3205 (4%)	0.004
Dogs and cats in compound	506/1075 (47%)	913/1983 (46%)	1419/3058 (46%)	0.59
Eats cassava	832 (74%)	1673 (80%)	2505 (75%)	<0.001
Eats soil	211 (19%)	167 (8%)	378 (12%)	<0.001
Bed net use	975 (87%)	1769 (85%)	2744 (86%)	0.12
Snores at night	253 (23%)	477 (23%)	730 (23%)	0.84
Deceased father	34/1051 (3%)	65/1967 (3%)	99/3018 (3%)	0.92

The risk factors questionnaire was administered to 3207 (46%) of the 7047 children screened, and included most acute seizures

*IQR* interquartile range

All *p* values > 0.01 are reported to 2 decimal places, while those <0.01 are reported to 3 decimal places

### Prevalence of acute seizures

There were 614 children screening for any seizures in stage I (Fig. 1), of whom 56 (9.1%) were treated for seizures in KCH, suggesting that the treatment gap for seizures is >90%. The total number of provoked seizures after accounting for attrition (between stages I and II) was 429 (261 from clinical history or hospital records, 145 from multiple imputation and 23 extrapolated from the assessment of screen negatives) (Fig. 1), which is an overall prevalence of 6.1 per 100 (95% CI: 5.5–6.8). The prevalence ratio of provoked seizures as a function of age-year was 1.10 (95% CI: 1.04–1.17) (Additional file 2: Figure S1). Of the 261 children with provoked seizures confirmed by a clinician, 126 (48%) met the definition for febrile seizures, while the remainder were acute symptomatic seizures. This translates to a prevalence of 2.9 per 100 (95% CI: 2.6–3.3) for febrile seizures and 3.2 (95% CI: 2.9–3.5) for acute symptomatic seizures based on the numbers in Fig. 1.

### Clinical and electroencephalographic features of acute seizures

The median age of onset of all provoked seizures was 13 months (interquartile range, IQR, 6–25). Most children experienced generalised tonic-clonic seizures (221/261 or 85%), few had multiple seizure types (17/261 or 7%), while seizure type could not be established in the remainder. Complex provoked seizures (focal, repetitive and/or prolonged) occurred in 240/261 (84%) of all seizures, in 114/135 (84%) of acute symptomatic seizures and in 106/126 (84%) of febrile seizures (Additional file 2: Figure S2). Provoked seizures preceded a history of epilepsy in 30/261 (11%) children. Of the 261 provoked seizures diagnosed by a clinician, 158 (61%) children agreed to undergo an EEG recording, with no differences in age (*p* = 0.08), sex (*p* = 0.77) or complex phenotypes (*p* = 0.83) between those with and without an EEG assessment. Abnormalities were detected in 43/158 (27%) of the EEGs and 22/43 (51%) of the abnormalities were focal. An abnormal EEG was neither correlated with focal seizure semiology (tetrachoric rho = 0.12, *p* = 0.42) nor with neurological or motor deficits (tetrachoric rho = 0.15, *p* = 1.00).

### Causes and risk factors of acute seizures

Among the identifiable causes of provoked seizures, falciparum malaria was the most important cause of provoked seizures (112/261 or 43%), followed by respiratory tract infections (68/261 or 26%). The causes were not statistically different between complex and simple provoked seizures (Additional file 2: Table S3). In the multivariable analysis, risk factors for provoked seizures included family history of febrile seizures (adjusted odds ratio (aOR) = 3.19; 95% CI: 2.03–5.01) and previous hospitalisation (aOR = 6.65; 95% CI: 4.60–9.63) (Additional file 2: Table S4).

### Distribution of CBCL scores by acute seizure status

**Fig. 2:**
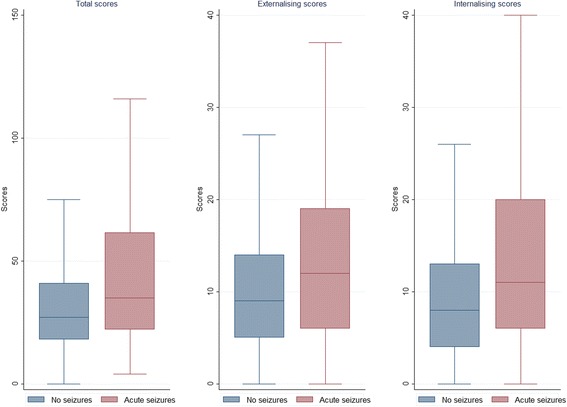
Distribution of behavioural and emotional problem scores by acute seizure status. The scores for behavioural and emotional problems were significantly higher in children with acute seizures than in those without seizures for total problems, externalising problems and internalising problems

Of the 3273 children who received the CBCL, 200 (6.1%) had provoked seizures while 26 (0.7%) had had provoked seizures preceding epilepsy. The mean and median scores were significantly higher in those with provoked seizures than those without seizures for total problems, externalising problems, internalising problems and for the six CBCL syndromes and five DSM-IV-oriented scales (Table 2 and Fig. 2). However, effect sizes were variable for comparisons between provoked seizures and those without these seizures, and appeared greatest for internalising problems (Cohen’s *d* = 0.44; 95% CI: 0.29–0.58) (Table 2).

**Table 2:** Effect sizes and distribution of behavioural and emotional scores between those with and without acute seizures

	Median scores (IQR)			Mean scores (SD)			
Behavioural and emotional scores	Number without seizures (*N* = 3073)	Acute seizures (*N* = 200)	*p* value^a^	Number of acute seizures(*N* = 3073)	Acute seizures (*N* = 200)	*p* value^b^	Cohen’s *d* (95% CI)
Total	27 (18–41)	35 (22–62)	<0.001	33.0 (22.5)	43.2 (27.1)	<0.001	0.44 (0.30–0.59)
Internalising	8 (4–13)	11 (6–20)	<0.001	10.0 (8.4)	13.8 (10.5)	<0.001	0.44 (0.29–0.58)
Externalising	9 (5–14)	12 (6–19)	<0.001	10.7 (7.7)	13.6 (8.9)	<0.001	0.38 (0.24–0.52)
CBCL syndromes							
Emotionally reactive	2 (1–3)	2 (1–5)	0.009	2.3 (2.4)	3.0 (3.1)	0.002	0.27 (0.13–0.41)
Anxious or depressed	2 (0–4)	3 (1–7)	<0.001	2.8 (2.7)	4.1 (3.6)	<0.001	0.43 (0.28–0.57)
Somatic problems	2 (1–4)	4 (2–6)	<0.001	3.0 (2.6)	4.3 (3.5)	<0.001	0.47 (0.32–0.62)
Withdrawn	1 (0–3)	2 (1–4)	0.009	1.9 (2.1)	2.4 (2.4)	0.001	0.24 (0.10–0.38)
Sleep problems	2 (1–3)	2 (1–4)	<0.001	2.2 (2.2)	2.9 (2.5)	<0.001	0.32 (0.17–0.46)
Attention problems	3 (2–5)	4 (2–5)	0.002	3.4 (2.1)	3.8 (2.1)	0.002	0.23 (0.08–0.37)
Aggressive behaviour	6 (3–10)	8 (4–15)	<0.001	7.3 (6.5)	9.8 (7.5)	<0.001	0.38 (0.24–0.52)
DSM-oriented scales							
Affective problems	1 (0–3)	2 (1–4)	<0.001	1.9 (2.4)	3.0 (2.9)	<0.001	0.43 (0.28–0.57)
Anxiety problems	4 (2–6)	5 (2–8)	<0.001	4.4 (3.5)	5.6 (4.0)	<0.001	0.32 (0.18–0.47)
Pervasive developmental problems	2 (1–4)	3 (2–5)	0.004	3.1 (2.9)	3.7 (3.3)	0.005	0.20 (0.06–0.35)
Attention deficit or hyperactivity problems	5 (3–7)	6 (4–8)	0.004	5.4 (2.9)	6.0 (3.0)	0.003	0.22 (0.07–0.36)
Oppositional defiant problems	2 (0–3)	3 (1–4)	<0.001	2.2 (2.3)	2.9 (2.4)	<0.001	0.32 (0.18–0.46)

All *p* values > 0.01 are reported to 2 decimal places, while those <0.01 are reported to 3 decimal places

^a^Mann–Whitney U test comparison of median behavioural and emotional scores

^b^Student’s *t*-test comparison of behavioural and emotional scores

*CI* confidence interval, *IQR* interquartile range, *DSM* Diagnostic and Statistical Manual of Mental Disorders, *SD* standard deviation

Effect sizes were a function of mean scores and standard deviation

The median total CBCL scores for children with acute symptomatic seizures (37; IQR: 23–63) and febrile seizures (35; IQR: 23–62) were higher than for those without seizures (27; IQR: 18–21); *p* < 0.001 for both comparisons. This difference in scores was consistent with the effect sizes (Cohen’s *d* = 0.59, 95% CI: 0.32–0.80, for acute symptomatic seizures) and (Cohen’s *d* = 0.35, 95% CI: 0.10–0.61, for febrile seizures).

### Prevalence of CBCL problems by acute seizure status

The crude prevalence of total CBCL problems was 27% (95% CI: 21–34%) for those with provoked seizures, 30% (95% CIs: 20–43%) for those with acute symptomatic seizures and 25% (95% CI: 15–38%) for those with febrile seizures; all being significantly greater than for those without seizures (11%; 95% CI: 11–12%; chi-squared *p* ≤ 0.001). The crude prevalence of externalising problems was significantly greater in provoked seizures (18%; 95% CI: 13–24%), in acute symptomatic seizures (23%; 95% CI: 14–35%) and in febrile seizures (22%; 95% CI: 12–35%) than in those without seizures (12%; 95% CI: 11–13%); chi-squared *p* < 0.001 for all comparisons. Among those with provoked seizures, total CBCL problems were not significantly increased in those with and without focal provoked seizures (35% vs 30%, *X*^2^ *p* = 0.41), neurological deficits (25% vs 39%, Fisher’s exact *p* value = 1.00) or abnormal EEG (11% vs 30%, Fisher’s exact *p* = 0.09).

### Independent association between CBCL problems and acute seizures

Provoked seizures were associated with total CBCL problems (adjusted risk ratio (aRR) = 1.92; 95% CI: 1.34–2.77), externalising problems (aRR = 1.82; 95% CI: 1.21–2.75) and internalising problems (aRR = 1.57; 95% CI: 1.22–2.02) after accounting for socio-demographic factors (e.g. child’s age) and other non-seizure confounders (e.g. perinatal complications) (Table 3). The associations for total CBCL problems were greater for acute symptomatic seizures (RR = 3.64; 95% CI: 1.94–6.84) than for febrile (aRR = 2.00; 95% CI: 1.02–3.92), which was supported by the ROR for the two associations (1.82; 95% CI: 1.31–2.53).

**Table 3:** Association of acute seizures with binary behavioural and emotional problems as the outcome/dependent variable

Behavioural and emotional problems	Number without acute seizures (*N* = 3073)	Acute seizures (200)	Adjusted RR (95% CI)^a^	AIC^a^	Adjusted RR (95% CI)^b^	AIC^b^	AIC^b^ < AIC^a^
Total problems	366 (12%)	54 (27%)	*2.24 (1.75–2.88)*	0.78	*2.40 (1.61–3.58)*	0.41	Yes
Externalising problems	302 (10%)	36 (18%)	*1.76 (1.28–2.41)*	0.67	*2.09 (1.40–3.13)*	0.41	Yes
Internalising problems	648 (21%)	80 (40%)	*1.92 (1.60–2.31)*	1.11	*1.99 (1.40–2.91)*	0.41	Yes
CBCL syndromes							
Emotionally reactive	299 (10%)	32 (16%)	*1.67 (1.19–2.33)*	0.67	1.38 (0.86–2.21)	0.41	Yes
Anxious or depressed	362 (12%)	55 (28%)	*2.38 (1.86–3.05)*	0.77	*2.18 (1.50–3.18)*	0.41	Yes
Somatic problems	622 (20%)	73 (37%)	*1.77 (1.45–2.15)*	1.08	*2.15 (1.52–3.05)*	0.41	Yes
Withdrawn	327 (11%)	33 (17%)	*1.58 (1.14–2.20)*	0.71	1.27 (0.78–2.05)	0.41	Yes
Sleep problems	100 (3%)	14 (7%)	*2.06 (1.19–3.57)*	0.30	*1.82 (1.00–3.35)*	0.41	Yes
Attention problems	461 (15%)	41 (21%)	*1.33 (1.00–1.76)*	0.89	*1.52 (1.04–2.23)*	0.41	Yes
Aggressive behaviour	166 (5%)	23 (12%)	*2.04 (1.36–3.07)*	0.44	*1.87 (1.19–2.94)*	0.41	Yes
DSM-oriented scales							
Affective problems	163 (5%)	26 (13%)	*2.41 (1.65–3.53)*	0.44	*2.05 (1.18–3.54)*	0.39	Yes
Anxiety problems	363 (12%)	45 (23%)	*1.91 (1.45–2.52)*	0.77	*1.64 (1.14–2.35)*	0.67	Yes
Pervasive developmental problems	157 (5%)	16 (8%)	1.59 (0.98–2.60)	0.42	0.85 (0.42–1.73)	0.37	Yes
Attention deficit or hyperactivity problems	146 (5%)	17 (8%)	*1.72 (1.06–2.77)*	0.40	1.38 (0.69–2.75)	0.34	Yes
Oppositional defiant problems	71 (2%)	5 (3%)	1.02 (0.42–2.51)	0.22	1.01 (0.31–3.30)	0.18	Yes

*AIC* Akaike’s information criterion, *CBCL* Child Behaviour Checklist, *CI* confidence interval, *RR* risk ratio

^a^Model one, in which RR is adjusted for child’s age and sex and region of residence. ^b^Model two, in which RR is adjusted for known non-seizure risk factors for behavioural and emotional problems namely pregnancy problems, perinatal complications, maternal age, maternal employment, house status and head injury, in addition to those adjusted in model one. The italicised RR reached a cut-off *p*-value of 0.05 or less, or their lower CI did not cross over 1

Behavioural and emotional problems were entered into the model as binary variables

In similar adjusted models, specific CBCL syndromes remained associated with provoked seizures, particularly anxious or depressed problems (aRR = 1.90; 95% CI: 1.34–2.70) and so were the DSM-IV-oriented scales, particularly affective problems (aRR = 2.05; 95% CI: 1.18–3.54) (Table 3). These associations remained significant even with continuous CBCL scores as the response variable (Additional file 2: Table S5). The proportion of the association between behavioural and emotional problems and provoked seizures mediated by a co-diagnosis of epilepsy was small (15.3%; 95% CI: 4.5–34.9%) (Additional file 2: Table S6).

### Risk factors for behavioural and emotional comorbidity of acute seizures

In those with provoked seizures, five risk factors were significantly associated with total CBCL problems in the multivariable model, notably family history of febrile seizures (RR = 3.36; 95% CI: 1.34–8.41). These five significant risk factors explained 30% of the variation in the model (Additional file 2: Table S7). In those with provoked seizures, nine risk factors were significantly associated with externalising problems in the multivariable model, among them repetitive provoked seizures (β = 0.36; 95% CI: 0.15–0.57). These nine significant risk factors explained 79% of the variation in the model (Additional file 2: Table S8). In those with provoked seizures, four risk factors were significantly associated with internalising problems in the multivariable model, among them focal provoked seizures (RR = 1.80; 95% CI: 1.05–3.08). These four significant risk factors explained 48% of the variation in the model (Additional file 2: Table S9).

## Discussion

This population-based study shows that the prevalence of acute seizures in this rural area of Kenya is high (6.1%) in children aged 1 year and that risk factors include family history of febrile seizures and previous hospitalisation, perhaps related to high levels of acute infections in this area. Acute seizures remained independently associated with behavioural and emotional problems after accounting for non-seizure confounders, being greater in acute symptomatic seizures than febrile seizures as would be expected. The most important risk factors for increased behavioural and emotional problems in children with acute seizures included family history of febrile seizures for total CBCL problems and externalising problems, repetitive acute seizures for externalising and internalising problems, and focal acute seizures for internalising problems.

### Prevalence, risk factors and features of acute seizures

The prevalence of febrile seizures (2.9%) was higher than found in other studies of febrile seizures, including Tanzania (2.1%) [7] and the United Kingdom (2.3%), but lower than in the United States of America (3.9%) [26]. The Tanzanian study was conducted within administrative boundaries in which vital statistics are updated less often than the demographic surveillance areas used in our study, which can potentially affect prevalence estimates. Most seizures with fever in Kilifi are acute symptomatic seizures [10], including in this study (3.2%), perhaps explaining the low prevalence of febrile seizures in our study compared to the American study. Some febrile seizures could be symptomatic, considering this study was in a malaria-endemic area, where parasites may sequester into the brain [10]. The prevalence estimates from our study confirm that hospital incidence [27] grossly underestimates the true prevalence of acute seizures in the community. The large treatment gap for acute seizures in this area should be addressed, since only 9% of those screening for seizures in stage I had ever sought treatment for seizures at the only referral hospital in this area.

Like studies from resource-rich countries [28], a family history of febrile seizures was documented in up to 40% of children with acute seizures, suggesting a genetic susceptibility or shared environmental factors. The association with previous hospitalisation likely points to febrile illnesses that cause acute seizure, e.g. falciparum malaria (identified as the most common cause of acute seizures in this study; 43%). Cassava consumption, which may be a marker of poverty, is associated with neuropsychiatric problems [29]. Our study was not designed to investigate the possible neurotoxicity of cassava.

The relatively lower age of onset, compared to other studies [3], reflects the susceptible period for the infectious causes of acute seizures in this area. Complex acute seizures were common (85%), comparable to previous studies in this area (84%) [13]. EEGs were abnormal in about 30%, suggesting a propensity to develop epilepsy [9], and the explanation of why acute seizures preceded epilepsy in 11% of children in this study. These features may affect mental health outcomes in children with acute seizures.

### Independent association between acute seizures and CBCL problems

Acute seizures remained associated with behavioural and emotional problems, externalising problems and internalising problems after accounting for potential demographic and non-seizure confounders. This was consistent with the frequency and effect size differences (using Cohen’s *d* statistics) between those with acute seizures and those without. Acute symptomatic seizures contributed significantly greater behavioural and emotional comorbidities than febrile seizures since the ROR and its corresponding lower confidence interval were greater than 1. A small proportion of the association (15%) was mediated by a co-diagnosis of epilepsy, a marker of underlying cortical damage. These associations do not imply a causal association, as this was a cross-sectional study, although we asked about behavioural and emotional problems in the past 2 months, when some children would have experienced their first acute seizures. Alternatively, acute seizures can presumably create anxiety in the child and the mother [30].

### Risk factors for behavioural and emotional problems in those with acute seizures

Multivariable models identified significant risk factors that explained the substantial variation in these models, e.g. up to 80% for externalising problems. These factors can explain the behavioural and emotional comorbidity of acute seizures in three ways: (i) direct biological effect, (ii) shared pathway and (iii) confounding or bias. A family history of febrile seizures (associated with total CBCL and externalising problems) suggests there is an underlying genetic susceptibility [31]. Repetitive or focal seizures can have a direct biological effect through damaging parts of the brain or changing the maturation of neuronal networks, possibly explaining the association between behavioural and emotional problems and acute seizures. Head injury and cognitive impairment could be markers of a shared pathway, e.g. underlying neurological damage [32] associated with both behavioural problems and acute seizures. The number of siblings can increase family or parental distress [33], thereby confounding the association between behavioural and emotional problems and acute seizures. Snoring, when caused by an upper airway obstruction, can have a direct hypoxic effect on the brain that manifests as behavioural and emotional problems in these children with seizures [14]. The role of eating soil in the behavioural and emotional comorbidity of acute seizures is unclear and requires further investigation, since the association could be due to residual confounding by the effects of heavy metals in soils, which may be associated with neurodevelopmental impairments [34].

### Strengths and limitations

To our knowledge, this is the largest epidemiology study of acute seizures and behavioural emotional problems in young children within the community in Africa using a robust demographic surveillance system. The two-stage methodology ensured that most acute seizures were diagnosed by a clinician. Behavioural and emotional problems were assessed with an internationally applied behavioural and emotional assessment tool (the CBCL), which was validated and adapted to the local population [19].

Attrition between stages I and II (addressed with multiple imputation) and the limited sampling of clinical assessment and EEG may affect the interpretation of results. Although we leveraged on a demographic surveillance that is linked to admissions to KCH to establish the acute seizure status of children who did not turn up for clinical evaluation in stage II, our demographic surveillance is not linked to other peripheral health facilities and private clinics, where some children may have been treated for acute seizures. The use of multiple imputation to account for attrition may introduce some bias if there were some unmeasured factors that could make failure to report for assessment occur at random. Maternal mental health status, quality of parenting, HIV status and family functioning may influence behavioural and emotional outcomes [30], but they were not assessed in our study.

## Conclusion

The prevalence of acute seizures in Kilifi was found to be higher than in many other parts of the world, and these seizures are independently associated with behavioural and emotional problems. Clinicians should identify and address behavioural and emotional problems in children presenting with acute seizures to improve outcomes. This can be achieved by training primary health-care workers (e.g. clinicians and nurses) on how to identify and manage behavioural and emotional problems in the context of acute seizures, using World Health Organization approved intervention guidelines such as the Mental Health Gap Action Programme [35]. This is important because this combination of problems has the potential to put these children’s long-term development at risk. Parenting programmes should be initiated to support parents of children who experience both behavioural and emotional problems and seizures. The association between acute seizures with behavioural and emotional problems may be explained in part by genetic factors, a hypothesis which should be investigated in future genetic studies. Longitudinal studies are, however, required to understand the direction of causality in the association between acute seizures and behavioural and emotional problems.

## Additional files

Additional file 1: Screening Questionnaire for Seizure disorders. (DOC 143 kb)

Additional file 2: **Table S1.** Definitions of seizures and medical terms; **Table S2.** Identification and classification of febrile and non-febrile causes of children with acute seizures based on the WHO's Integrated Management of Childhood Infections (IMCI); **Table S3.** Causes of acute seizures diagnosed by a clinician according to phenotype in preschool children; **Table S4.** Bivariate and multivariable results for the factors associated with acute seizures diagnosed by a clinician; **Table S5.** Association of acute seizures with continuous behavioural and emotional scores as the outcome/dependent variable; **Table S6.** Proportion of total effect of acute seizures on CBCL problems mediated by a co-diagnosis of epilepsy; **Table S7.** Risk factors associated with total behavioural and emotional comorbidity of acute seizures; **Table S8.** Risk factors for externalising problems in children with acute seizures; **Table S9.** Risk factors for internalising problems in children with acute seizures; **Figure S1.** Prevalence of acute seizures in preschool children by age group; **Figure S2.** The overlap of focal, repetitive and prolonged phenotypes of acute seizures in 221 preschool children. (DOCX 96 kb)

## Abbreviations

CBCL: Child Behaviour Checklist
CI: Confidence interval
DSM-IV: Diagnostic and Statistical Manual of Mental Disorders IV
EEG: Electroencephalography
GLM: Generalised linear model
IQR: Interquartile range
KCH: Kilifi County Hospital
KHDSS: Kilifi Health and Demographic Surveillance System
OR: Odds ratio
ROR: Ratio of risk ratios
RR: Risk ratio
SD: Standard deviation
