# Supplementary material for: Prevalence, risk factors and behavioural and emotional comorbidity of acute seizures in young Kenyan children: a population-based study
Source: BMC Med. 2018 Mar 7;16:35. doi: 10.1186/s12916-018-1021-y (PMC5840716; doi:10.1186/s12916-018-1021-y)
Supplement: Supplementary file 1 — Screening Questionnaire for Seizure disorders. (DOC 143 kb) [file 12916_2018_1021_MOESM1_ESM.doc]

|  | **Today’s Date** | | | | | | | | | Y | Y | Y | Y |  | M | M |  | D | D | | --- | --- | --- | --- | --- | --- | --- | --- | --- | --- | | | | | | | | | | | | | | | |  | | | | | | **TDATE** | | |
| --- | --- | --- | --- | --- | --- | --- | --- | --- | --- | --- | --- | --- | --- | --- | --- | --- | --- | --- | --- | --- | --- | --- | --- | --- | --- | --- | --- | --- | --- | --- | --- | --- | --- | --- | --- | --- | --- | --- | --- | --- | --- | --- |
|  | Study Number | |  |  |  |  |  |  |  |  |  |  | | --- | --- | --- | --- | --- | --- | --- | --- | --- | --- | | | | | | | | | | | | | | | | | **STNO** | | | | | | | | | | | | | | |
|  | PID | | | | | | 1 | 1 | 1 | 1 | 1 | 1 | | --- | --- | --- | --- | --- | --- | | | | | | | | | | | | | | | **PID** | | | | | | | | | | | | |
|  | RESID | | | | | | 1 | 1 | 1 | 1 | 1 | 1 | 1 | | --- | --- | --- | --- | --- | --- | --- | | | | | | | | | | | | | | | **RESID** | | | | | | | | | | | | |
|  | Name and Surname | | | | | **NAME** | | | | | | | | | | | | | | | | | | | | | | | | | | |
|  | Age (estimated if date of birth unknown)   | Y | Y | Y | Y |  | M | M |  | D | D | | --- | --- | --- | --- | --- | --- | --- | --- | --- | --- | | | | | | | | | **DOB**   | 1 | 1 | | --- | --- | | | | | | | | | | | | | | | | **AGE** | | | | | | | | |
|  | Gender **M**ale/ **F**emale | | | | | | | | | 1 | | --- | | | | | | | | | | | | | | | |  | | | | | | **SEX** | | |
| **For all children in the study aged 0-6 years/ unable respondents.** | | | | | | | | | | | | | | | | | | | | | | | | | | | | | |  | | |
| Mother/Guardian’s Name  **MOTHNAME** | | | | | | | | | | | | | | | | | | | | | | | | | | | | | | | | |
| Who will answer questions about the index? | | | **1** = Self  **2** =Self and other  **3**= The Index’s mother  **4** = The Index’s father | | | | | | | **5** = Index’s grandmother  **6** = Index’s sibling  **7** = Another relative  **8** = Other | | | | | | | | | | | | **QCHILD**   | 1 | | --- | | | | | | | | | | |  |
| **For all children in the study aged 0-6 years/ unable respondents.** | | | | | | | | | | | | | | | | | | | | | | | | |  | | | | | | | |
| Is the informant one who mainly takes care of the Index patient? | | | | | | | | | | | | | **Y**es/ **N**o   | 1 | | --- | | | | | | | | | | | |  | | | | **INFORM** | | | | |
|  | Answer Yes(**Y**) or No (**N**) | | | | | | | | | | | |  | | | | | | | | | | |  | | | |  | | | | |
| 1. Vidze mwanao adzangwe kufitika hangu azhalwe?   *Has your child ever had a fit?* | | | | | | | | | | | | **Y**es/ **N**o | | | | | | | | | | 1 | | --- | | | | | **HAVEFITS** | | | | | | | |
| 1. Mutu adzangwe kukwambira kukala mwanao wafitika?   *Has someone ever told you that your child had fits?* | | | | | | | | | | | | **Y**es/ **N**o | | | | | | | | | | 1 | | --- | | | | | **SOMEFITS** | | | | | | | |
| 1. Udzangwe kwambirwa kukala mwanao ana vitsala hedu kufitika kwa vitsala?   *Have you ever been told that your child had epileptic fits?* | | | | | | | | | | | | **Y**es/ **N**o | | | | | | | | | | 1 | | --- | | | | | **EPIFITS** | | | | | | | |
| 1. Mwanao adzangwe kukala na vipindi ambazho anagwa hotsi na kungamiza fahamu *Has your child ever had attacks in which (s)he fall to the ground with loss of consciousness?* | | | | | | | | | | | | **Y**es/ **N**o | | | | | | | | | | 1 | | --- | | | | | **CONSC** | | | | | | | |
| 1. Mwanao adzangwe kugwa hotsi bila sababu yoyosi na   *Has your child ever fallen to the ground without reason and experienced:* | | | | | | | | | | | | | | | | | | | | | | |  | |  | | | | | | | |
| Kupigwa ni zhoyo *Twitching* | | | | | | | | **Y**es/ **N**o | | | | | | | | | | | | 1 | | --- | | | | | | | **TWITCH** | | | | | | | |
| Kuthethemwa ni mikono yosi hedu magulu gosi bila kudziunya *Shaking of the arms or legs without control* | | | | | | | | **Y**es/ **N**o | | | | | | | | | | | | 1 | | --- | | | | | | | **SHAKING** | | | | | | | |
| Na kudzikodzolera *Wetting him(er)self* | | | | | | | | **Y**es/ **N**o | | | | | | | | | | | | 1 | | --- | | | | | | | **WETTING** | | | | | | | |
| Kuluma luthimi *Biting of the tongue* | | | | | | | | **Y**es/ **N**o | | | | | | | | | | | | 1 | | --- | | | | | | | **BITING** | | | | | | | |
| 1. Udzangwe kwambirwa ni dakitari kukala mwanao ana vitsala hedu kufitika kwa vitsala?   *Have you ever been told by a doctor that your child has epileptic fits?* | | | | | | | | **Y**es/ **N**o | | | | | | | | | | | | 1 | | --- | | | | | | | **DRHAVE** | | | | | | | |
| 1. Kufitika kungine kwa sababishwa ni mwiri moho   *Did some seizures occur with a febrile illness or fever?* | | | | | | | | **Y**es/ **N**o | | | | | | | | | | | | 1 | | --- | | | | | | | **SZ_FILL** | | | | | | | |
| **If yes to any of the above 7 questions:** | | | | | | | | | | | | | | | | | | | | | | | | | | | | | | | | |
| I. Wafitika rini mwanzo? *When did the seizures start?* | | | | | Y | Y | Y | Y |  | M | M |  | D | D | | --- | --- | --- | --- | --- | --- | --- | --- | --- | --- | | | | | | | | | | | | | | | | **SZ_START** | | | | | | | | | | | | | |
| II.Wafitika rini mwisho? *When was the last seizure?* | | | | | | | | | | | | | | | Y | Y | Y | Y |  | M | M |  | D | D | | --- | --- | --- | --- | --- | --- | --- | --- | --- | --- | | | | | | **LAST_SZ** | | | | | | | | | | | | | |
| III.Kufitika kwahusisha sehemu mwenga ya mwiri?  *Did the seizures involve one body part?* | | | | | | | | | | | | | | **Y**es/ **N**o | | | | | | 1 | | --- | | | | | | | | | **SZ_FOC** | | | | | |
| IV.Kufitika kwahusisha sehemu zosi za mwiri ?  *Did the seizures involve all body parts?* | | | | | | | | | | | | | | **Y**es/ **N**o | | | | | | 1 | | --- | | | | | | | | | **SZ_GEN** | | | | | |
| V. Kufitika kwahala muda wani  *How long did the seizures last* | | | | | | | | | | | | | | **Y**es/ **N**o | | | | | | 1 | | --- | | | | | | | | | **SZ_DUR** | | | | | |
| VI.Kufitika kwahala Zaidi ya dakikat 30  *Did the seizures last ≥30 minutes* | | | | | | | | | | | | | | **Y**es/ **N**o | | | | | | 1 | | --- | | | | | | | | | **SZ_SE1** | | | | | |
| VII. Kufitika kwahala Zaidi ya muda wa kuchemusha nyungu ya matsere  *Did the seizures last longer than boiling a pot of maize* | | | | | | | | | | | | | | **Y**es/ **N**o | | | | | | 1 | | --- | | | | | | | | | **SZ_SE2** | | | | | |
| VIII. Kufitika kwahala Zaidi ya muda wa ibada kanisani  *Did the seizures last longer than a sermon in church or mosque* | | | | | | | **Y**es/ **N**o | | | | | | | | | | | | | 1 | | --- | | | | | | | | | | | **SZ_SE3** | | | |
| IX. Kufitika na/au wo mwiri moho watibiwa sipitali?  *Were the seizures and/or the febrile illness treated at a hospital?* | | | | | | | | | | | **Y**es/ **N**o | | | | | | | | | 1 | | --- | | | | | | | | | | | **SZ_HOS** | | | |
| X. Unahumira madawa gogosi ga kufitika kwa wakathi uno?  *Are you currently using any drugs for convulsions?* | | | | | | | | | | | **Y**es/ **N**o | | | | | | | | | 1 | | --- | | | | | | | | | | | **MEDIC** | | | |
| XI. Udzangwe kwenda kwa muganga kwa kulagulwa kufitika?  *Have you ever visited a traditional healer for treatment of seizures?* | | | | | | | | | | | **Y**es/ **N**o | | | | | | | | | 1 | | --- | | | | | | | | | | | **TRAD_H** | | | |
| **Family and Community seizure information** | | | | | | | | | | | | | | | |  | | | | | | | | | |  | | |  | | | |
|  | Vidze hana mutu yeyosi kwenye familiya ambaye anafitika  *Does any other member of the family have seizures?* | | | | | | | | | | | | | | | **Y**es/ **N**o | | | | | | | | | | | 1 | | --- | | | | **FAM_SZ** | | | |
|  | Ikikala ahaha ni hani? If*Who?* | | | | | | | | | | | | | | |  | | | | | | | | | |  | | | |  | | |
| | **Name** | | **Relationship** | **Agincourt No.** | | --- | --- | --- | --- | | **1.** |  |  | | 1 | 1 | 1 | 1 | 1 | 1 | | --- | --- | --- | --- | --- | --- | | | **2.** |  |  | | 1 | 1 | 1 | 1 | 1 | 1 | | --- | --- | --- | --- | --- | --- | | | **3.** |  |  | | 1 | 1 | 1 | 1 | 1 | 1 | | --- | --- | --- | --- | --- | --- | | | | | | | | | | | | | | | | | | | | | | | | | | | | | | | | | | |
|  | Unamanya muhoho yeyosi (9 months-6 years) ambaye anafitika *Do you know of other child(9-6years)with seizures?*  **Y**es/ **N**o | | | | | | | | | | | | | | | 1 | | --- | | | | | | | | | | | | **ANYB_SZ** | | | | | | |
|  | Ikikala a haho ni hani? *Who?* | | | | | | | | | | | | | | | | | | | | | | | | | | | | | | | |
| | **Name** | | **Relationship** | **Agincourt No.** | | --- | --- | --- | --- | | **1.** |  |  | | 1 | 1 | 1 | 1 | 1 | 1 | | --- | --- | --- | --- | --- | --- | | | **2.** |  |  | | 1 | 1 | 1 | 1 | 1 | 1 | | --- | --- | --- | --- | --- | --- | | | **3.** |  |  | | 1 | 1 | 1 | 1 | 1 | 1 | | --- | --- | --- | --- | --- | --- | | | | | | | | | | | | | | | | | | | | | | | | | | | | | | | | | | |
| **Interviewer:** | | | | |  | | | | | | | | | | | | | |  | | | | | | | | | | |  | | |
| Answer the question below by ticking the appropriate answer. An individual will only be referred if the answer to ANY of the above 7 questions **YES** | | | | | | | | | | | | | | | | | | | | | | | | | | | | | | | | |
|  | Should this person be referred for further evaluation? **Y**es/ **N**o | | | | | | | | | | | | | | | 1 | | --- | | | | | | | | | | | | **REF_EVAL** | | | | | | |
| **Interviewer:** | | | | | | | | | | | | | | | | | | | | | | | | | | | | | | | | |
|  | Has the information sheet been read to the respondent? | | | | | | | **Y**es/ **N**o   | 1 | | --- | | | | | | | | | | | | | | | | | **COM_SHT** | | | | | | | | |
|  | Has the respondent consented to participate in the study? | | | | | | | **Y**es/ **N**o   | 1 | | --- | | | | | | | | | | | | | | | | | **CONSENT** | | | | | | | | |
|  | If NO consent is given, what are the reasons given for the decline? | | | | | | | | | | | | | | | | | | | | | | |  | | | | | |  | | |
|  | **REAS_NOC1**   | 1 | |  | | --- | | | --- | --- | --- | | 2 | |  | | --- | | | 3 | |  | | --- | |   **REAS_NOC2**  **REAS_NOC3** | | | | | | | | | | | | | | | | | | | | | | | | | | | | | | | |
| **Interviewer:** | | | | | | | | | | | | | | | | |  | | | | | | | | |  | | | | |  | |
| Answer the questions below by ticking the appropriate answer. An individual will only be referred as a control for Phase III if they answer **NO** to all the first 6 questions. Please proceed to recruit the controls as follows; | | | | | | | | | | | | | | | | | | | | | | | | | | | | | | | | |
| Are you willing to be a control in this study (please explain what a control is if need be)? **Y**es/ **N**o | | | | | | | | | | | | | | | | | | 1 | | --- | | | | | | | | | | **CTRL_ST** | | | | | | |
| If the individual responds YES to the above 2 question, please notify them of the intention to invite them to the assessment centre for assessments. | | | | | | | | | | | | | | | | | | | | | | | | | | | | | | | | |
|  | Has this person agreed to come for assessment? **Y**es/ **N**o | | | | | | | | | | | | | | | | |  | | --- | | | | | | | | | | **AGREED** | | | | | | |
| **Sign if you have checked that the form is complete .** | | | | | | | | | | | | | | | | | | | | | | | | | | | | | | | | |
