# Supplementary material for: Prevalence, risk factors and behavioural and emotional comorbidity of acute seizures in young Kenyan children: a population-based study
Source: BMC Med. 2018 Mar 7;16:35. doi: 10.1186/s12916-018-1021-y (PMC5840716; doi:10.1186/s12916-018-1021-y)
Supplement: Supplementary file 2 — Table S1. Definitions of seizures and medical terms; Table S2. Identification and classification of febrile and non-febrile causes of children with acute seizures based on the WHO's Integrated Management of Childhood Infections (IMCI); Table S3. Causes of acute seizures diagnosed by a clinician according to phenotype in preschool children; Table S4. Bivariate and multivariable results for the factors associated with acute seizures diagnosed by a clinician; Table S5. Association of acute seizures with continuous behavioural and emotional scores as the outcome/dependent variable; Table S6. Proportion of total effect of acute seizures on CBCL problems mediated by a co-diagnosis of epilepsy; Table S7. Risk factors associated with total behavioural and emotional comorbidity of acute seizures; Table S8. Risk factors for externalising problems in children with acute seizures; Table S9. Risk factors for internalising problems in children with acute seizures; Figure S1. Prevalence of acute seizures in preschool children by age group; Figure S2. The overlap of focal, repetitive and prolonged phenotypes of acute seizures in 221 preschool children. (DOCX 96 kb) [file 12916_2018_1021_MOESM2_ESM.docx]

**Additional file 2**

**Table S1. Definitions of seizures and medical terms**

| **Term** | **Definition** |
| --- | --- |
| Acute seizures | Acute seizures were defined as seizures occurring with a febrile illness or associated with a recorded fever (>38.5◦ C). |
| Epilepsy | Epilepsy was defined as a history of two unprovoked seizures with those having one of the seizures in the past one year considered to have active epilepsy according to ILAE recommendations |
| Focal seizures | Seizures that involved one body part, and generalised seizures those involving the entire body |
| Prolonged seizures | Seizures were considered prolonged if they lasted 15 minutes or more |
| Repetitive seizures | Two or more acute seizures in the same illnesses or within 24 hours |
| Complex seizures | Acute seizures that were either focal, prolonged or repetitive |
| Malnutrition | Weight for age (WAZ) or height for age (HAZ) z-score value of ≤-2. |

**All seizures definitions were based on ILAE recommendations. Febrile temperatures were only confirmed using a thermometer for children admitted to the hospital with seizures, but for other acute seizures it was based on parental report of fever.**

**Table S2: Identification and classification of febrile and non-febrile causes of children with acute seizures based on the WHO’s Integrated Management of Childhood Infections (IMCI)**

| **Febrile Illness/cause** | **Description** |
| --- | --- |
| Malaria | Any fever without running nose or respiratory tract symptoms, since Kilifi is a high risk area |
| Very severe febrile disease e.g. meningitis | Fever with stiff neck and crying a lot or irritability |
| Upper respiratory tract infection (RTI) | Fever with running nose and a mild cough |
| Lower RTI (pneumonia) | Fever with severe cough, fast breathing (>40/min)and chest indrawing |
| Diarrhoea e.g. gastroenteritis | Fever or no fever with 3 or 4 loose stools in 24 hours, lethargy or not able to feed properly |
| Ear infections | Fever with ear discharge, ear pain and/or swelling behind ear |
| Measles | History of fever and eye discharge and/or mouth ulcers with or without respiratory and ear infections or diarrhoea or rash |
| Undetermined cause | Neither of the above |

**NB: For infants: history of presence of a bulging of fontanelle and a red umbilicus draining pus may suggest febrile illness e.g. bacterial infections**

**Table S3. Causes of acute seizures diagnosed by a clinician according to phenotype in preschool children**

| **Diagnosis** | **Focal phenotype (N=79)** | **Repetitive phenotype (N=121)** | **Prolonged phenotype (N=156)** | **All Complex phenotype (N=221)** | **All Simple seizures (N=40)** | **All acute seizures (N=261)** | **P-value*** |
| --- | --- | --- | --- | --- | --- | --- | --- |
| Malaria | 35  (44%) | 56  (46%) | 68  (44%) | 96  (43%) | 16 (40%) | 112 (43%) | 0.686 |
| Respiratory tract infections | 19  (24%) | 32  (26%) | 43  (28%) | 57  (26%) | 11 (28%) | 68 (26%) | 0.821 |
| Gastroenteritis/diarrhoea | 3  (4%) | 10  (8%) | 11  (7%) | 17  (8%) | 1  (3%) | 18  (7%) | 0.324 |
| Meningitis | 1  (1%) | 3  (2%) | 2  (1%) | 3  (1%) | 0  (0%) | 3  (1%) | 1 |
| Measles | 0  (0%) | 1  (1%) | 0  (0%) | 1  (<1%) | 0  (0%) | 1  (<1%) | 1 |

***Pearson’s Chi-square comparison of the causes between the complex phenotypes versus simple phenotypes. The causes are not mutually exclusive. Fishers exact was used to compared infrequent observations (<5 in a cell)**

**Table S4. Bivariate and multivariable results for the factors associated with acute seizures diagnosed by a clinician**

|  | **Bivariate analysis** | | | **Multivariable analysis** | | |
| --- | --- | --- | --- | --- | --- | --- |
| **Factor** | **No seizures (N=2907)** | **Acute seizures (N=245)** | **Odds ratio (95%CI)** | **p-value** | **Odds ratio (95%CI)** | **p-value** |
| **Child’s age in months: median(IQR)** | 46 (30-62) | 50 (34-67) | **1.01 (0.99-1.01)** | **0.054** | 1.00 (0.99-1.01) | 0.446 |
| **Child’s sex** | 1452 (50) | 141 (55%) | **1.17 (0.90-1.52)** | **0.239** | 1.02 (0.72-1.45) | 0.901 |
| **Maternal age** | 30 (25-35) | 27 (24-33) | 0.99 (0.98-1.02) | 0.734 | - | - |
| **Mother’s marital status** |  |  |  |  |  |  |
| Single | 142 (5%) | 8 (3%) | 1.00 |  | 1.00 |  |
| Separated/divorced | 178 (6%) | 18 (7%) | **1.79 (0.76-4.25)** | **0.183** | 2.25 (0.76-6.61) | 0.142 |
| Widowed | 51 (2%) | 8 (3%) | **2.75 (0.99-7.80)** | **0.052** | 2.57 (0.68-9.82) | 0.162 |
| Married | 2536 (87%) | 211 (86%) | 1.48 (0.71-3.05) | 0.292 | 1.45 (0.59-3.58) | 0.442 |
| **Mother’s religion** |  |  |  |  |  |  |
| None | 714 (25%) | 70 (29%) | 1.00 |  | 1.00 |  |
| Traditional | 167 (6%) | 2 (1%) | **0.12 (0.03-0.50)** | **0.004** | **0.09 (0.01-0.81)** | **0.031** |
| Islam | 346 (12%) | 30 (12%) | 0.88 (0.57-1.38) | 0.590 | 1.13 (0.61-2.07) | 0.704 |
| Christian | 1680 (58%) | 148 (58%) | 0.87 (0.64-1.17) | 0.354 | 1.10 (0.73-1.65) | 0.661 |
| **Mother’s education level** |  |  |  |  |  |  |
| None | 867/2904 (30%) | 76/241 (32%) | 1.00 |  | - | - |
| Primary | 1812/2904 (62%) | 147/241 (61%) | 0.93 (0.69-1.23) | 0.599 | - | - |
| Secondary | 183/2904 (6%) | 15/241 (6%) | 0.94 (0.53-1.66) | 0.819 | - | - |
| Tertiary | 42/2904 (1%) | 3/241 (1%) | 0.81 (0.25-2.69) | 0.7371 | - | - |
| **Mother’s ethnicity** |  |  |  |  |  |  |
| None-mijikenda | 149 (5%) | 13/243 (5%) | 1.00^b^ |  | - | - |
| Other mijikenda | 173 (6%) | 16/243 (7%) | 1.06 (0.49-2.28) | 0.881 | - | - |
| Kauma | 273 (9%) | 34/243 (14%) | 1.43 (0.73-2.79) | 0.298 | - | - |
| Chonyi | 847 (29%) | 81/243 (33%) | 1.10 (0.60-2.01) | 0.769 | - | - |
| Giriama | 1465 (50%) | 99/243 (41%) | 0.77 (0.42-1.41) | 0.406 | - | - |
| **Economic activity: mother** | 2086/2902 (72%) | 149/244 (61%) | **0.61 (0.47-0.80)** | **<0.0001** | **0.64 (0.44-0.92)** | **0.016** |
| **Economic activity: father** | 2487/2687 (93%) | 209/228 (92%) | 0.88 (0.54-1.45) | 0.625 | - | - |
| **Number of children: median (IQR)** | 4 (3-6) | 3 (2-6) | 1.02 (0.97-1.07) | 0.458 | - | - |
| **Water availability** |  |  |  |  |  |  |
| Infrequent | 594 (20%) | 21 (9%) | 1.00^b^ |  | 1.00 |  |
| Weekly | 318 (11%) | 61 (25%) | **5.43 (3.24-9.07)** | **<0.0001** | **2.42 (1.19-4.94)** | **0.016** |
| Daily | 643 (22%) | 21 (9%) | 0.92 (0.50-1.71) | 0.801 | 1.20 (0.56-2.56) | 0.642 |
| Always | 1352 (47%) | 142 (58%) | **2.97 (1.85-4.75)** | **<0.001** | **2.09 (1.11-3.92)** | **0.022** |
| **House status** |  |  |  |  |  |  |
| Dilapidated | 136/2906 (5%) | 41/244 (17%) | 1.00^b^ |  | 1.00 |  |
| Major repairs needed | 245/2906 (8%) | 53/244 (22%) | 0.72 (0.45-1.13) | 0.156 | 0.95 (0.48-1.87) | 0.879 |
| Incompletely build | 77/2906 (3%) | 7/244 (3%) | **0.30 (0.13-0.70)** | **0.006** | 0.39 (0.12-1.11) | 0.075 |
| Minor/no repairs needed | 2448/2906 (84%) | 143/244 (57%) | **0.19 (0.13-29)** | **<0.0001** | **0.46 (0.26-0.83)** | **0.009** |
| **Toilet type** |  |  |  |  |  |  |
| Bush/none | 819/2906 (28%) | 64/244 (26%) | **1.00^b^** |  | 1.00 |  |
| Traditional pit | 1647/2906 (57%) | 130/244 (54%) | 1.01 (0.74-1.38) | 0.950 | 0.89 (0.59-1.33) | 0.564 |
| Ventilated pit | 272/2906 (9%) | 25/244 (10%) | 1.18 (0.73-1.90) | 0.509 | 0.98 (0.50-1.91) | 0.954 |
| Flush | 168/2906 (6%) | 24/244 (10%) | **1.83 (1.11-3.01)** | **0.017** | 1.25 (0.64-2.45) | 0.516 |
| **Livestock owned** |  |  |  |  |  |  |
| None | 1364 (47%) | 116 (47%) | 1.00 |  |  |  |
| <5 | 595 (20%) | 44 (18%) | 0.87 (0.61-1.25) | 0.447 | - | - |
| >5 | 948 (33%) | 85 (35%) | 1.05 (0.79-1.41) | 0.722 | - | - |
| Pregnancy problems | 189 (7%) | 35/242 (15%) | **2.43 (1.65-3.58)** | **<0.0001** | 1.34 (0.78-2.28) | 0.286 |
| Mother’s age at birth | 18 (17-20) | 18 (17-20) | 1.00 (0.96-1.03) | 0.784 | - | - |
| Delivery at home | 1793/2906 (62%) | 151 (62%) | 1.00 (0.76-1.31) | 0.983 | - | - |
| Adverse perinatal events | 73/2906 (3%) | 13/242 (5%) | **2.20 (1.20-4.04)** | **0.011** | 0.74 (0.33-1.68) | 0.477 |
| Family history of seizures | 277 (10%) | 97% (40%) | **6.28 (4.66-8.45)** | **<0.0001** | **2.52 (1.64-3.88)** | **<0.0001** |
| Family history of febrile seizures | 223/2906 (8%) | 84 (34%) | **6.22 (4.68-8.27)** | **<0.0001** | **3.19 (2.03-5.01)** | **<0.0001** |
| Previous hospitalisation | 265/2903 (9%) | 111/237 (47%) | **8.77**  **(6.60-11.65)** | **<0.0001** | **6.65 (4.60-9.63)** | **<0.0001** |
| Head injury | 54/2905 (2%) | 10 (4%) | **2.25 (1.13-4.47)** | **0.021** | 0.91 (0.36-2.25) | 0.883 |
| Dogs and cats in compound | 1264/2823 (45%) | 125/185 (68%) | **2.57 (1.87-3.53)** | **<0.0001** | **1.65 (1.14-2.37)** | **0.008** |
| Eats cassava | 2225 (77%) | 234 (96%) | **6.52**  **(3.54-12.01)** | **<0.0001** | **5.15 (2.28-11.63)** | **<0.0001** |
| Eats soil | 327 (11%) | 40/244 (16%) | **1.55 (1.08-2.21)** | **0.017** | 1.25 (0.78-2.00) | 0.360 |
| Bednet use | 2472 (85%) | 227 (93%) | **2.35 (1.42-3.89)** | **0.001** | **1.97 (1.03-3.75)** | **0.040** |
| Snores at night | 612/2906 (21%) | 101/244 (41%) | **2.65 (2.02-3.47)** | **<0.0001** | **1.60 (1.10-2.32)** | **0.013** |
| Deceased father | 90/2734 (3%) | 9/234 (4%) | 1.17 (0.58-2.36) | 0.651 | **-** | **-** |

^a^Includes univariate risk factors with a p-value ≤0.25 in a logistic regression model with child’s age and sex.

^b^Statistically significant linear trend for the categories. Cells marked with dash (-) represents the variables which did not qualify for the multivariable analysis. PAF=population-attributable fraction; CI=confidence interval. Approximately 16/261 (6%) children with clinically confirmed acute seizures did not receive the risk factor questionnaire and were excluded from the risk factor analysis. Approximately 55 children with a risk factor questionnaire (but without acute seizures) had other seizures disorders e.g. epilepsy and hence were excluded from the comparison group in the risk factor analysis. A Bonferroni corrected p-value of 0.001 should be deemed significant in the multivariable analysis.

**Table S5. Association of acute seizures with continuous behavioural and emotional scores as the outcome/dependent variable**

| **Behavioural and emotional scores** | **Adjusted βC (95%CI)^a^** | **AIC^a^** | **Adjusted βC (95%CI)^b^** | **AIC^b^** | **AIC^b^<AIC^a^** |
| --- | --- | --- | --- | --- | --- |
| Total problems | **0.27**  **(0.18-0.36)** | 1.96 | **0.21**  **(0.09-0.33)** | 1.78 | Yes |
| Externalising problems | **0.25**  **(0.14-0.36)** | 2.31 | **0.23**  **(0.09-0.36)** | 2.19 | Yes |
| Internalising problems | **0.29**  **(0.17-0.42)** | 2.41 | **0.25**  **(0.10-0.41)** | 2.33 | Yes |
| **CBCL syndromes** | | | | | |
| Emotionally-reactive | **0.15**  **(0.03-0.27)** | 2.10 | 0.08  (-0.08-0.23) | 2.01 | Yes |
| Anxious-depressed | **0.26**  **(0.14-0.39)** | 2.33 | **0.21**  **(0.05-0.37)** | 2.25 | Yes |
| Somatic problems | **0.25**  **(0.14-0.36)** | 2.03 | **0.29**  **(0.15-0.44)** | 1.98 | Yes |
| Withdrawn | **0.11**  **(0.00-0.23)** | 1.97 | 0.09  (-0.05-0.24) | 1.93 | Yes |
| Sleep problems | **0.11**  **(0.01-0.21)** | 1.81 | 0.07  (-0.06-0.20) | 1.74 | Yes |
| Attention problems | 0.08  (-0.02-0.17) | 1.84 | 0.10  (-0.01-0.22) | 1.80 | Yes |
| Aggressive behaviour | **0.29**  **(0.16-0.41)** | 2.53 | **0.22**  **(0.07-0.38)** | 2.43 | Yes |
| **DSM-IV-oriented scales** | | | | | |
| Affective problems | **0.22**  **(0.10-0.34)** | 2.15 | **0.22**  **(0.07-0.38)** | 2.43 | No |
| Anxiety problems | **0.20**  **(0.08-0.31)** | 2.16 | 0.13  (-0.01-0.28) | 2.10 | Yes |
| Pervasive developmental problems | **0.15**  **(0.04-0.26)** | 2.19 | 0.10  (-0.04-0.24) | 2.11 | Yes |
| Attention deficit/hyperactivity problems | **0.09**  **(0.00-0.18)** | 1.88 | 0.06  (-0.05-0.18) | 1.80 | Yes |
| Oppositional defiant problems | **0.17**  **(0.07-0.28)** | 2.05 | **0.14**  **(0.01-0.28)** | 1.97 | Yes |

AIC=Akaike’s information criterion; βC=beta coefficient; CI=confidence interval; ^a^Model one; ^b^Model two; βC^a^ is adjusted for child’s age and sex and region of residence while βC^b^ is adjusted for known non-seizure risk factors for behavioural and emotional problems namely pregnancy problems, perinatal complications, maternal age, maternal employment, house status and head injury; in addition to those adjusted in βC^a^. All behavioural and emotional scores were entered into the model as continuous variables.

**Table S6. Proportion of total effect of acute seizures on CBCL problems mediated by a co-diagnosis of epilepsy.**

| **Behavioural and emotional problems** | **Total effect**  **βC (95%CI)** | **Direct effect βC (95%CI)** | **Indirect effects βC (95%CI)** | **Proportion mediated in % (95%CI)** |
| --- | --- | --- | --- | --- |
| Total problems | 0.26 (0.13-0.38) | 0.22 (0.12-0.31) | 0.04 (0.01-0.07) | 15.3 (4.5-34.9) |
| Externalising problems | 0.25 (0.11-0.40) | 0.22 (0.10-0.34) | 0.03 (0.01-0.06) | 12.0 (2.5-31.2) |
| Internalising problems | 0.28 (0.11-0.45) | 0.23 (0.10-0.36) | 0.05 (0.01-0.09) | 17.8 (6.1-36.9) |

Behavioural and emotional problems entered into the Sobel-Goodman mediation tests as continuous scores. βC=Beta coefficient; CI=confidence interval

**Table S7. Risk factors associated with total behavioural and emotional comorbidity of acute seizures**

|  |  |  | **Binary behavioural and emotional problems** | | **Continuous behavioural and emotional scores** | |
| --- | --- | --- | --- | --- | --- | --- |
| **Risk factor** | **No behavioural problems** | **Behavioural problems** | **Adjusted RR (95%CI)^a^** | **Multivariable RR (CI)** | **Adjusted β coefficient (95%CI)^b^** | **Multivariable β coefficient (95%CI)** |
| Mother’s age in years: median (IQR) | 27 (24-34) | 26 (23-35) | 1.00  (0.97-1.04) | - | -0.00  (-0.02, 0.01) | - |
| Sociodemographic information | N=115 | N=41 |  |  |  |  |
| **Mother’s marital status** |  |  |  | - |  |  |
| Single | 6 (5%) | 1 (2%) | 1.00 | 1.00 | 0.00 |  |
| Separated/divorced | 6 (5%) | 7 (17%) | **4.00**  **(0.62-25.92)** | Large SE | 0.05  (-0.49, 0.59) | - |
| Widowed | 4 (3%) | 1 (2%) | 1.45  (0.12-18.02) | Large SE | -0.16  (-0.71, 0.39) | - |
| Married | 99 (86%) | 32 (78%) | 1.75  (0.28-10.97) | Large SE | -0.15  (-0.50, 0.19) | - |
| **Mother’s religion** |  |  |  |  |  |  |
| None | 35 (30%) | 10 (24%) | 1.00 | - | 0.00 | 0.00 |
| Traditional | 2 (2%) | 0 (0%) | - | - | 0.11  (-0.50, 0.71) | - |
| Islam | 13 (11%) | 6 (15%) | 1.42  (0.59-3.44) | - | **0.23**  **(-0.10, 0.56)** | 0.10 (-0.31, 0.50) |
| Christian | 65 (57%) | 25 (61%) | 1.30  (0.68-2.46) | - | 0.10  (-0.14, 0.33) | -0.03  (-0.27, 0.21) |
| **Mother’s education level** |  |  |  |  |  |  |
| None | 39 (34%) | 11 (27%) | 1.00 | - | 0.00 | 0.00 |
| Primary | 64 (56%) | 27 (66%) | 1.35  (0.73-2.50) | - | **0.20**  **(-0.03, 0.44)** | -0.05  (-0.43, 0.69) |
| Secondary | 9 (8%) | 2 (5%) | 0.87  (0.22-3.34) | - | 0.17  (-0.25, 0.58) | 0.13  (-0.43, 0.69) |
| Tertiary | 3 (3%) | 1 (2%) | 1.04  (0.18-5.91) | - | 0.13  (-0.42, 0.68) | -0.37  (-1.07, 0.32) |
| Economic activity: mother | 78 (68%) | 23 (56%) | **0.67**  **(0.39-1.14)** | 0.69  (0.37-1.30) | **-0.21**  **(-0.42, 0.01)** | -0.22  (-0.50, 0.06) |
| Economic activity: father | 98/108 (91%) | 36/39 (92%) | 1.17  (0.41-3.32) | - | 0.05  (-0.36, 0.46) | - |
| Number of children: median (IQR) | 4 (2-6) | 4 (2-6) | 1.01  (0.91-1.12) | - | -0.02  (-0.06, 0.02) | - |
| **Socioeconomic status and household information** | N=115 | N=41 |  |  |  |  |
| **Water availability** |  |  |  |  |  |  |
| Infrequent | 10/115 (9%) | 5/41 (12%) | 1.00 | 1.00 | 1.00 | - |
| Weekly | 23/115 (20%) | 9/41 (22%) | 0.87  (0.35-2.14) | 0.58  (0.17-1.94) | 0.01  (-0.33, 0.35) | - |
| Daily | 24/115 (21%) | 3/41 (7%) | **0.34**  **(0.10-1.21)** | **0.13**  **(0.03-0.64)** | -0.23  (-0.60, 0.14) | - |
| Always | 58/115 (50%) | 24/41 (59%) | 0.91  (0.42-1.97) | 0.60  (0.21-1.73) | -0.05  (-0.37, 0.27) | - |
| **House status** |  |  |  |  |  |  |
| Dilapidated | 18 (16%) | 9 (22%) | 1.00 | - | 1.00 | 0.00 |
| Major repairs needed | 16/115 (14%) | 9 (22%) | 1.16  (0.54-2.47) | - | 0.15  (-0.19, 0.50) | -0.16  (-0.50, 0.17) |
| Incompletely build | 7 (6%) | 0 (0%) | - | - | **-0.23**  **(-0.55, 0.09)** | -0.12  (-0.60, 0.37) |
| Minor/no repairs needed | 74 (64%) | 23 (56%) | 0.74  (0.39-1.40) | - | -0.07  (-0.31, 0.16) | -0.16  (-0.50, 0.18) |
| **Toilet type** |  |  |  |  |  |  |
| Bush/none | 29 (25%) | 12 (29%) | 1.00 | - | 1.00 |  |
| Traditional pit | 65 (57%) | 20 (49%) | 0.79  (0.43-1.45) | - | -0.07  (-0.31, 0.17) | - |
| VIP pit | 9 (8%) | 5 (12%) | 1.22  (0.52-2.88) | - | 0.01  (-0.42, 0.45) | - |
| Flush | 12 (10%) | 4 (10%) | 0.81  (0.31-2.12) | - | 0.01  (-0.37, 0.38) | - |
| **Livestock owned** |  |  |  |  |  |  |
| None | 52 (45%) | 23 (56%) | 1.00 | - | 1.00 | 0.00 |
| <5 | 20 (17%) | 6 (15%) | 0.75  (0.35-1.61) | - | **-0.19**  **(-0.48, 0.08)** | 0.06 (-0.23, 0.35) |
| >5 | 43 (37%) | 12 (29%) | 0.74  (0.40-1.37) | - | **-0.19**  **(-0.42, 0.04)** | -0.05  (-0.44, 0.33) |
| **Pregnancy and birth history** |  |  |  |  |  |  |
| Pregnancy problems | 16 (14%) | 7 (17%) | 1.22  (0.61-2.45) | - | -0.05  (-0.39, 0.28) | - |
| Mother’s age at birth | 18 (17-20) | 18 (17-19) | **0.95**  **(0.87-1.03)** | 0.94  (0.86-1.03) | -0.02  (-0.05, 0.02) | - |
| Delivery at home | 72 (63%) | 23 (56%) | 0.83  (0.49-1.38) | - | -0.10  (-0.31, 0.11) | - |
| Adverse perinatal events | 6 (5%) | 4 (10%) | 1.54  (0.68-3.51) | - | 0.15  (-0.17, 0.48) | - |
| **Medical history** | N=115 | N=41 |  |  |  |  |
| Previous hospitalisation | 54 (47%) | 17 (41%) | 0.84  (0.49-1.44) | - | -0.08  (-0.28, 0.12) | - |
| Head injury | 4 (3%) | 4 (10%) | **2.02**  **(0.91-4.49)** | **2.46**  **(1.00-6.10)** | **0.48**  **(0.15, 0.81)** | 0.25 (-0.29, 0.80) |
| Dogs and cats in compound | 65 (57%) | 18 (44%) | **0.70**  **(0.41-1.21)** | 0.60  (0.29-1.25) | -0.07  (-0.27, 0.14) | - |
| Eats cassava | 105 (91%) | 40 (98%) | 2.98  (0.46-19.32) | 0.42  (0.08-2.12) | 0.17  (-0.13, 0.46) | - |
| Eats soil | 14 (12%) | 11 (27%) | **2.14**  **(1.22-3.76)** | **2.19**  **(1.04-4.59)** | **0.53**  **(0.25, 0.80)** | 0.26 (-0.05, 0.56) |
| Bednet use | 102 (89%) | 35 (85%) | 0.86  (0.40-1.86) | - | **0.19**  **(-0.13, 0.51)** | 0.02 (-0.45, 0.50) |
| Snores at night | 40 (35%) | 19 (46%) | **1.46**  **(0.87-2.47)** | 1.38  (0.79-2.41) | **0.16**  **(-0.06, 0.37)** | **0.23 (0.02, 0.45)** |
| Deceased father | 5 (5%) | 1 (3%) | 0.65  (0.10-4.24) | - | 0.11  (-0.36, 0.57) | - |
| **Seizure factors** | N=146 | N=54 |  |  |  |  |
| Family history of seizures | 36/115 (31%) | 19/41 (46%) | **1.55**  **(0.91-2.63)** | 1.30  (0.65-2.61) | **0.24**  **(0.04, 0.44)** | 0.04 (-0.23, 0.31) |
| Family history of febrile seizures | 76/115 (66%) | 36/41 (88%) | **2.91**  **(1.20-7.05)** | **3.36**  **(1.34-8.41)** | **0.31**  **(0.11, 0.51)** | **0.39 (0.06, 0.72)** |
| Prolonged seizures | 84/120 (70%) | 29/41 (71%) | 0.94  (0.50-1.7) | - | -0.02  (-0.24, 0.20) | - |
| Repetitive seizures | 37/128 (29%) | 17/51 (33%) | **1.49**  **(0.94-2.36)** | 1.18  (0.57-2.43) | **0.14**  **(-0.05, 0.34)** | 0.22 (-0.02, 0.46) |
| Focal seizures | 39 (27%) | 21 (39%) | 1.22  (0.75-1.99) | - | 0.09  (-0.11, 0.29) | - |
| All complex seizures | 110 (75%) | 40 (74%) | 0.97  (0.58-1.62) | - | -0.06  (-0.27, 0.16) | - |
| Abnormal EEG | 14/59 (24%) | 2/22 (9%) | **0.41 (0.10-1.57)** | Large SE | **-0.27 (-0.55, 0.03)** | Large SE |
| Epilepsy co-diagnosis | 15 (10%) | 11 (20%) | **1.69**  **(0.99-2.86)** | 1.73  (0.92-3.26) | **0.22**  **(-0.03, 0.48)** | 0.06 (-0.26, 0.37) |
| **Medical examination** | N=84 | N=39 |  |  |  |  |
| Developmental delays | 5/80 (6%) | 6/34 (18%) | **2.03**  **(1.11-3.72)** | 1.27  (0.65-2.50) | **0.49**  **(0.18, 0.81)** | -0.20  (-0.61, 0.21) |
| Neurological/motor deficits | 15 (18%) | 8 (21%) | 1.36  (0.67-2.75) | - | **0.30**  **(-0.04, 0.65)** | 0.10 (-0.24, 0.44) |
| Intellectual disability | 4 (5%) | 3 (8%) | 1.31  (0.57-2.98) | - | **0.30**  **(0.10, 0.50)** | 0.59 (0.12, 1.06) |
| Peer-relationship problems | 5/78 (6%) | 3/34 (9%) | 1.40  (0.52-3.82) | - | **0.38**  **(-0.04, 0.79)** | -0.32  (-0.74, 0.11) |
| Sensation problems | 3 (4%) | 1 (3%) | 0.72  (0.12-4.50) | - | 0.10  (-0.64, 0.85) | - |
| Malnutrition | 3 (4%) | 1 (3%) | 0.85  (0.14-5.13) | - | -0.29  (-0.96, 0.37) | - |

Highlighted adjusted RR or β and multivariable RR or β reached the cut-off p-value of ≤0.25 and ≤0.05, respectively. ^a^Model one; ^b^Model two; RR^a^ and β^a^ are adjusted for child’s age and sex and region of residence. CI=confidence interval; IQR=interquartile range; RR=risk ratio; SE=standard error

**Table S8. Risk factors for externalising problems in children with acute seizures**

|  | **Externalising problems** | | **Binary externalising problems** | | **Continuous externalising scores** | |
| --- | --- | --- | --- | --- | --- | --- |
| **Factors** | **No** | **Yes** | **Adjusted RR (95%CI)** | **Multivariable RR (95%CI)** | **Adjusted β coefficient (95%CI)** | **Multivariable β coefficient (95%CI)** |
| Mother’s age in years: median (IQR) | 27 (24-32) | 29 (23-35) | 1.01 (0.97-1.05) | - | 0.00 (-0.01, 0.02) | - |
| Sociodemographic information | N=124 | N=32 |  |  |  |  |
| **Mother’s marital status** |  |  |  |  |  |  |
| Single | 6 (5%) | 1 (3%) | 1.00 | 1.00 | 0.00 | - |
| Separated/divorced | 7 (6%) | 6 (19%) | **3.97 (0.50-31.32)** | 5.95 (0.94-37.58) | 0.06 (-0.54, 0.66) | - |
| Widowed | 4 (3%) | 1 (3%) | 1.82 (0.12-27.50) | 2.63 (0.19-36.54) | -0.15 (-0.85, 0.54) | - |
| Married | 107 (86%) | 24 (75%) | 1.50 (0.20-10.95) | 1.13 (0.23-5.55) | -0.15 (-0.56, 0.26) | - |
| **Mother’s religion** |  |  |  |  |  |  |
| None | 39 (31%) | 6 (19%) | 1.00 | 1.00 | 0.00 | 0.00 |
| Traditional | 1 (1%) | 1 (3%) | **4.25 (0.82-22.24)** | 18.35 (0.79-425.72) | **0.53 (0.00, 1.06)** | Large SE |
| Islam | 13 (10%) | 6 (19%) | **2.25 (0.81-6.27)** | 1.93 (0.49-7.56) | 0.13 (-0.29, 0.54) | -0.11 (-0.64, 0.41) |
| Christian | 71 (57%) | 19 (59%) | **1.76 (0.78-3.99)** | 2.26 (0.21-23.85) | 0.09 (-0.17, 0.35) | -0.09 (-0.34, 0.15) |
| **Mother’s education level** |  |  |  |  |  |  |
| None | 40 (32%) | 10 (31%) | 1.00 | 1.00 | 0.00 | 0.00 |
| Primary | 72 (58%) | 19 (59%) | 1.05 (0.54-2.06) | 0.65 (0.30-1.42) | 0.13 (-0.15, 0.41) | -0.04 (-0.30, 0.23) |
| Secondary | 10 (8%) | 1 (3%) | 0.53 (0.07-3.88) | 0.26 (0.02-4.33) | 0.27 (-0.24, 0.78) | -0.36 (-0.18, 0.90) |
| Tertiary | 2 (2%) | 2 (2%) | **2.80 (0.82-9.54)** | 2.26 (0.21-23.88) | 0.38 (-0.30, 1.07) | -0.62 (-1.28, 0.05) |
| Economic activity: mother | 84 (68%) | 17 (53%) | **0.59 (0.32-1.10)** | 0.55 (0.24-1.26) | **-0.17 (-0.44, 0.10)** | -0.01 (-0.30, 0.28) |
| Economic activity: father | 107/118 (91%) | 27/29 (93%) | 1.32 (0.35-4.96) | - | 0.02 (-0.41, 0.45) | - |
| Number of children: median (IQR) | 4 (2-6) | 4 (3-7) | 1.02 (0.90-1.16) | - | **-0.04 (-0.08, 0.01)** | **0.12 (0.06, 0.17)** |
| **Socioeconomic status and household information** | N=124 | N=32 |  |  |  |  |
| **Water availability** |  |  |  |  |  |  |
| Infrequent | 10 (8%) | 5 (16%) | 1.00 | 1.00 | 0.00 | 0.00 |
| Weekly | 24 (19%) | 8 (25%) | 0.88 (0.36-2.18) | **0.32 (0.11-0.99)** | 0.05 (-0.37, 0.47) | -0.04 (-0.48, 0.39) |
| Daily | 25 (20%) | 2 (6%) | **0.25 (0.06-1.11)** | **0.12 (0.03-0.33)** | -**0.46 (-0.94, 0.02)** | **-0.46 (-0.91, -0.01)** |
| Always | 65 (52%) | 17 (53%) | 0.70 (0.32-1.53) | **0.27 (0.09-0.81)** | -0.08 (-0.45, 0.29) | **-0.37 (-0.71, -0.02)** |
| **House status** |  |  |  |  |  |  |
| Dilapidated | 22 (18%) | 5 (16%) | 1.00 | - | 0.00 | - |
| Major repairs needed | 17 (14%) | 8 (25%) | 1.82 (0.69-4.84) | - | 0.24 (-0.18, 0.66) | - |
| Incompletely build | 7 (6%) | 0 (0%) | Large SE |  | -0.02 (-0.40, 0.36) | - |
| Minor/no repairs needed | 78 (63%) | 19 (59%) | 1.15 (0.49-2.72) | - | -0.12 (-0.42, 0.18) | - |
| **Toilet type** |  |  |  |  |  |  |
| Bush/none | 32 (26%) | 9 (28%) | 1.00 | - | 0.00 | - |
| Traditional pit | 70 (56%) | 15 (47%) | 0.74 (0.36-1.54) | - | -0.10 (-0.39, 0.19) | - |
| VIP pit | 9 (7%) | 5 (16%) | 1.56 (0.62-3.95) | - | 0.11 (-0.36, 0.58) | - |
| Flush | 13 (10%) | 3 (9%) | 0.77 (0.24-2.40) | - | -0.11 (-0.60, 0.38) | - |
| **Livestock owned** |  |  |  |  |  |  |
| None | 56 (45%) | 19 (59%) | 1.00 | 1.00 | 0.00 | 0.00 |
| <5 | 21 (17%) | 5 (16%) | 0.76 (0.33-1.77) | 0.40 (0.13-1.20) | **-0.24 (-0.57, 0.09)** | 0.23 (-0.12, 0.58) |
| >5 | 47 (38%) | 8 (25%) | **0.62 (0.29-1.33)** | 0.93 (0.38-2.32) | **-0.22 (-0.49, 0.05)** | 0.04 (-0.26, 0.33) |
| **Pregnancy and birth history** |  |  |  |  |  |  |
| Pregnancy problems | 16 (13%) | 7 (22%) | **1.77 (0.86-3.66)** | 1.26 (0.36-4.43) | -0.09 (-0.49, 0.31) | - |
| Mother’s age at birth | 18 (17-20) | 18 (16-20) | 0.98 (0.90-1.07) | - | -0.01 (-0.05, 0.03) | - |
| Delivery at home | 80 (65%) | 15 (47%) | **0.59 (0.32-1.09)** | 0.95 (0.42-2.15) | **-0.24 (-0.48, 0.01)** | **-0.29 (-0.52, -0.07)** |
| Adverse perinatal events | 8 (6%) | 2 (6%) | 0.91 (0.24-3.46) | - | **0.26 (-0.05, 0.58)** | -0.11 (-0.53, 0.31) |
| **Medical history** | N=124 | N=32 |  |  |  |  |
| Previous hospitalisation | 57 (46%) | 14 (44%) | 0.93 (0.50-1.73) | - | -0.12 (-0.36, 0.13) | - |
| Head injury | 5 (4%) | 3 (9%) | **2.17 (0.79-6.02)** | 2.09 (0.16-26.59) | **0.70 (0.44, 0.97)** | 0.06 (-0.45, 0.57) |
| Dogs and cats in compound | 70 (56%) | 13 (41%) | **0.60 (0.32-1.14)** | 0.43 (0.17-1.08) | **-0.16 (-0.40, 0.08)** | -0.10 (-0.37, 0.18) |
| Eats cassava | 70 (56%) | 13 (41%) | 1.12 (0.31-3.97) | - | 0.14 (-0.28, 0.56) | - |
| Eats soil | 18 (15%) | 7 (22%) | **1.75 (0.86-3.58)** | 1.40 (0.41, 4.76) | **0.56 (0.28,0.83)** | **0.55 (0.21, 0.89)** |
| Bednet use | 108 (87%) | 29 (91%) | 1.70 (0.54-5.31) | - | **0.30 (-0.09, 0.70)** | 0.44 (-0.01, 0.87) |
| Snores at night | 46 (37%) | 13 (41%) | 1.18 (0.63-2.20) | - | 0.10 (-0.15, 0.36) | - |
| Deceased father | 6/120 (5%) | 0/28 (0%) | Large SE | - | 0.03 (-0.44, 0.50) | - |
| **Seizure factors** | **N=164** | **N=36** |  |  |  |  |
| Family history of seizures | 38/124 (31%) | 17/32 (53%) | **1.96 (1.05-3.65)** | **3.26 (1.58-6.73)** | **0.30 (0.06, 0.54)** | 0.16 (-0.10, 0.42) |
| Family history of febrile seizures | 84/124 (68%) | 28/32 (88%) | **2.79 (1.03-7.54)** | 1.21 (0.39-3.75) | **0.40 (0.15, 0.66)** | 0.31 (-0.03, 0.64) |
| Prolonged seizures | 97/137 (71%) | 16/24 (67%) | 0.85 (0.38-1.92) | - | 0.09 (-0.18, 0.35) | - |
| Repetitive seizures | 42 (26%) | 18 (50%) | **2.32 (1.31-4.10)** | 1.47 (0.68-3.15) | **0.24 (0.01, 0.46)** | **0.36 (0.15, 0.57)** |
| Focal seizures | 42/145 (29%) | 12/34 (35%) | 1.40 (0.75-2.61) | **-** | **-**0.10 (-0.36, 0.16) | - |
| All complex seizures | 124 (76%) | 26 (72%) | 0.96 (0.50-1.83) | - | -0.10 (-0.32, 0.12) | - |
| Abnormal EEG | 13/60 (22%) | 3/21 (14%) | 0.68 (0.23-2.03) | - | **-0.49 (-0.91, -0.07)** | Large SE |
| Epilepsy co-diagnosis | 19 (12%) | 7 (19%) | **1.58 (0.76-3.30)** | 0.96 (0.46-2.02) | 0.18 (-0.13, 0.48) | - |
| **Medical examination** | N=92 | N=31 |  |  |  |  |
| Developmental delays | 5/86 (6%) | 6/28 (21%) | **2.66 (1.50-4.71)** | 1.27 (0.32-5.03) | **0.39 (-0.03, 0.82)** | **-0.63 (-1.08, -0.17)** |
| Neurological/motor deficits | 16 (17%) | 7 (23%) | **1.77 (0.88-3.56)** | 2.04 (0.47-8.92) | 0.05 (-0.34, 0.44) | - |
| Intellectual disability | 4 (4%) | 3 (10%) | 1.56 (0.71-3.46) | - | **0.37 (-0.01, 0.74)** | **0.49 (0.15, 0.82)** |
| Academic difficulties | 3/58 (5%) | 2/17 (12%) | 1.79 (0.66-4.81) | - | **0.55 (0.24, 0.87)** | **1.01 (0.31, 1.72)** |
| Peer-relationship problems | 5/83 (6%) | 3/29 (10%) | **1.80 (0.67-4.84)** | 1.83 (0.51-6.54) | **0.43 (-0.02, 0.89)** | -0.24 (-0.86, 0.37) |
| Sensation problems | 3 (3%) | 1 (3%) | 0.77 (0.12-5.13) | - | 0.09 (-0.59, 0.77) | - |
| Malnutrition | 4 (4%) | 0 (0%) | Large SE | - | -0.29 (-0.94, 0.37) | - |

Highlighted adjusted RR or β and multivariable RR or β reached the cut-off p-value of ≤0.25 and ≤0.05, respectively. ^a^Model one; ^b^Model two; RR^a^ and β^a^ are adjusted for child’s age and sex and region of residence. CI=confidence interval; IQR=interquartile range; RR=risk ratio; SE=standard error

**Table S9. Risk factors for internalising problems in children with acute seizures**

|  | **Internalising problems** | | **Binary internalising problems** | | **Continuous internalising scores** | |
| --- | --- | --- | --- | --- | --- | --- |
| **Factors** | **No** | **Yes** | **Adjusted RR (95%CI)** | **Multivariable RR (95%CI)** | **Adjusted β coefficient (95%CI)** | **Multivariable β coefficient (95%CI)** |
| Mother’s age in years: median (IQR) | 30 (25-35) | 28 (24-35) | 0.99 (0.97-1.02) | - | -0.01 (-0.02, 0.01) | - |
| **Mother’s marital status** |  |  |  |  |  |  |
| Single | 4/96 (4%) | 3/60 (5%) | 1.00 | - | 0.00 | - |
| Separated/divorced | 6/96 (6%) | 7/60 (12%) | 1.39 (0.53-3.60) | - | 0.15 (-0.65, 0.96) | - |
| Widowed | 2/96 (2%) | 3/60 (5%) | 1.64 (0.57-4.72) | - | 0.01 (-0.94, 0.96) | - |
| Married | 84/96 (88%) | 47/60 (78%) | 0.93 (0.40-2.15) | - | -0.01 (-0.60, 0.57) | - |
| **Mother’s religion** |  |  |  |  |  |  |
| None | 28/96 (29%) | 17/60 (28%) | 1.00 | - | 0.00 | - |
| Traditional | 2/96 (2%) | 0 | - | - | -0.75 (-2.17, 0.67) | - |
| Islam | 12/96 (13%) | 7/60 (12%) | 0.98 (0.48-1.98) | - | 0.18 (-0.25, 0.60) | - |
| Christian | 54/96 (56%) | 36/60 (60%) | 1.15 (0.73-1.80) | - | 0.08 (-0.22, 0.37) | - |
| **Mother’s education level** |  |  |  |  |  |  |
| None | 33/96 (34%) | 17/60 (28%) | 1.00 | 1.00 | 0.00 | 0.00 |
| Primary | 51/96 (53%) | 40/60 (67%) | **1.32 (0.85-2.07)** | 1.33 (0.71-2.48) | **0.23 (-0.07, 0.53)** | 0.20 (-0.22, 0.63) |
| Secondary | 9/96 (9%) | 2/60 (3%) | 0.61 (0.16-2.29) | 0.44 (0.09-2.19) | 0.11 (-0.35, 0.56) | -0.13 (-0.87, 0.61) |
| Tertiary | 3/96 (3%) | 1/60 (2%) | 0.70 (0.13-3.88) | 1.80 (0.34-9.43) | -0.01 (-0.67, 0.66) | -0.44 (-1.37, 0.50) |
| Economic activity: mother | 69/96 (72%) | 32/60 (53%) | **0.61 (0.41-0.90)** | 0.61 (0.35-1.08) | **-0.28 (-0.54, -0.03)** | **-0.35 (-0.70, -0.01)** |
| Economic activity: father | 82/91 (90%) | 52/56 (93%) | 1.24 (0.52-2.94) | - | 0.15 (-0.37, 0.68) | - |
| Number of children: median (IQR) | 4 (3-6) | 4 (3-6) | 1.00 (0.93-1.09) | - | -0.01 (-0.06, 0.04) | - |
| **Socioeconomic status and household information** |  |  |  |  |  |  |
| **Water availability** |  |  |  |  |  |  |
| Infrequent | 10/96 (10%) | 5/60 (8%) | 1.00 |  | 0.00 | - |
| Weekly | 19/96 (20%) | 13/60 (22%) | 1.41 (0.64-3.10) | - | -0.15 (-0.56, 0.27) | - |
| Daily | 21/96 (22%) | 6/60 (10%) | 0.72 (0.27-1.92) | - | -0.11 (-0.51, 0.29) | - |
| Always | 46/96 (48%) | 36/60 (60%) | 1.45 (0.71-2.93) | - | 0.00 (-0.39, 0.39) | - |
| **House status** |  |  |  |  |  |  |
| Dilapidated | 15/96 (16%) | 12/60 (20%) | 1.00 | 1.00 | 0.00 | 0.00 |
| Major repairs needed | 9/96 (9%) | 16/60 (27%) | **1.53 (0.93-2.52)** | 1.74 (0.74-4.09) | **0.34 (-0.10, 0.77)** | -0.22 (-0.75, 0.31) |
| Incompletely build | 6/96 (6%) | 1/60 (2%) | **0.30 (0.04-2.08)** | 0.82 (0.13-5.26) | -0.20 (-0.64, 0.23) | -0.45 (-1.01, 0.11) |
| Minor/no repairs needed | 66/96 (69%) | 31/60 (52%) | 0.77 (0.47-1.25) | 1.13 (0.49-2.63) | 0.02 (-0.32, 0.35) | -0.36 (-0.79, 0.07) |
| **Toilet type** |  |  |  |  |  |  |
| Bush/none | 24/96 (25%) | 17/60 (28%) | 1.00 | - | 0.00 | - |
| Traditional pit | 52/96 (54%) | 33/60 (55%) | 0.89 (0.58-1.39) | - | -0.07 (-0.37, 0.24) | - |
| VIP pit | 9/96 (9%) | 5/60 (8%) | 0.82 (0.37-1.86) | - | -0.07 (-0.54, 0.40) | - |
| Flush | 11/96 (11%) | 5/60 (8%) | 0.72 (0.32-1.58) | - | 0.00 (-0.44, 0.43) | - |
| **Livestock owned** |  |  |  |  |  |  |
| None | 43/96 (45%) | 32/60 (53%) | 1.00 |  | 0.00 | - |
| <5 | 17/96 (18%) | 9/60 (15%) | 0.82 (0.46-1.44) | - | -0.11 (-0.45, 0.22) | - |
| >5 | 36/96 (38%) | 19/60 (32%) | 0.86 (0.55-1.34) | - | -0.10 (-0.38, 0.19) | - |
| **Pregnancy and birth history** |  |  |  |  |  |  |
| Pregnancy problems | 15/96 (16%) | 8/60 (13%) | 0.96 (0.53-1.73) | - | 0.04 (-0.32, 0.40) | - |
| Mother’s age at birth | 18 (17-20) | 18 (16-20) | **0.94 (0.88-1.01)** | 0.98 (0.89-1.06) | -0.03 (-0.07, 0.01) | - |
| Delivery at home | 59/96 (61%) | 36/60 (60%) | 0.99 (0.66-1.46) | - | -0.02 (-0.28, 0.24) | - |
| Adverse perinatal events | 5/96 (5%) | 5/60 (8%) | 1.28 (0.64-2.55) | - | -0.07 (-0.58, 0.46) | - |
| **Seizure factors** |  |  |  |  |  |  |
| Family history of seizures | 32/96 (33%) | 23/60 (38%) | 1.09 (0.73-1.63) | - | 0.10 (-0.18, 0.38) | - |
| Family history of febrile seizures | 60/96 (63%) | 52/60 (87%) | **2.64 (1.38-5.04)** | 2.61 (0.88-7.70) | **0.34 (0.06, 0.61)** | 0.26 (-0.17, 0.68) |
| Prolonged seizures | 72/101 (71%) | 41/60 (68%) | 0.90 (0.58-1.39) | - | **-0.17 (-0.47, 0.12)** | 0.28 (-0.21, 0.76) |
| Repetitive seizures | 31/120 (26%) | 29/80 (36%) | **1.31 (0.93-1.84)** | 1.15 (0.63-2.07) | **0.23 (-0.02, 0.47)** | **0.53 (0.23, 0.83)** |
| Focal seizures | 29/108 (27%) | 25/71 (35%) | **1.33 (0.92-1.93)** | **1.80 (1.05-3.08)** | **0.34 (0.09, 0.58)** | 0.33 (-0.06, 0.72) |
| All complex seizures | 92/120 (77%) | 58/80 (73%) | 0.90 (0.62-1.31) | - | -0.12 (-0.40, 0.16) | - |
| Abnormal EEG | 13/46 (28%) | 3/36 (9%) | 0.38 (0.13-1.09) | large SE | -0.36 (-0.76, 0.03) | large SE |
| Epilepsy co-diagnosis | 12/120 (10%) | 14/80 (18%) | **1.40 (0.92-2.11)** | 1.60 (0.83-3.07) | 0.18 (-0.20, 0.56) | - |
| **Medical examination** |  |  |  |  |  |  |
| Developmental delays | 4/64 (6%) | 7/50 (14%) | **1.58 (1.01-2.45)** | 0.64 (0.29-1.44) | **0.54 (0.20, 0.88)** | -0.35 (-0.82, 0.12) |
| Neurological/motor deficits | 13/67 (19%) | 10/56 (18%) | 1.16 (0.69-1.95) | - | 0.20 (-0.23, 0.64) | - |
| Intellectual disability | 2/67 (3%) | 5/56 (9%) | **1.48 (0.95-2.32)** | 1.60 (0.64-3.96) | **0.23 (-0.02, 0.48)** | **0.62 (0.01, 1.24)** |
| Academic difficulties | 2/40 (5%) | 3/35 (9%) | 1.23 (0.56-2.70) | - | **0.26 (-0.06, 0.59)** | 0.14 (-0.41, 0.69) |
| Peer-relationship problems | 3/62 (5%) | 5/50 (10%) | **1.69 (0.96-2.97)** | 1.60 (0.77-3.35) | **0.44 (-0.04, 0.92)** | -0.36 (-0.93, 0.21) |
| Sensation problems | 2/67 (3%) | 2/56 (4%) | 0.92 (0.30-2.79) | - | 0.17 (-0.73, 1.07) | - |
| Malnutrition | 3/67 (5%) | 1/56 (2%) | 0.60 (0.10-3.63) | - | -0.46 (-1.45, 0.53) | - |
| **Medical history** |  |  |  |  |  |  |
| Previous hospitalisation | 45/96 (47%) | 26/60 (43%) | 0.91 (0.61-1.35) | - | -0.05 (-0.30, 0.20) | - |
| Head injury | 3/96 (3%) | 5/60 (8%) | **1.81 (0.94-3.45)** | 2.16 (0.82-5.67) | **0.32 (-0.19, 0.83)** | -0.48 (-1.74, 0.78) |
| Dogs and cats in compound | 52/96 (54%) | 31/60 (52%) | 0.96 (0.64-1.43) | - | -0.11 (-0.37, 0.14) | - |
| Eats cassava | 86/96 (90%) | 59/60 (98%) | **4.37 (0.70-27.17)** | Large SE | 0.12 (-0.17, 0.42) | - |
| Eats soil | 11/96 (11%) | 14/60 (23%) | **1.84 (1.21-2.79)** | 1.85 (0.93-3.68) | **0.46 (0.08, 0.85)** | -0.29 (-0.74, 0.16) |
| Bednet use | 86/96 (90%) | 51/60 (85%) | 0.93 (0.54-1.59) | - | 0.16 (-0.27, 0.61) | - |
| Snores at night | 35/96 (36%) | 24/60 (40%) | 1.12 (0.75-1.68) | - | 0.09 (-0.19, 0.36) | - |
| Deceased father | 2/92 (2%) | 4/56 (7%) | **1.82 (1.06-3.12)** | 0.45 (0.19-1.06) | 0.22 (-0.54, 0.99) | - |

Highlighted adjusted RR or β and multivariable RR or β reached the cut-off p-value of ≤0.25 and ≤0.05, respectively. ^a^Model one; ^b^Model two; RR^a^ and β^a^ are adjusted for child’s age and sex and region of residence. CI=confidence interval; IQR=interquartile range; RR=risk ratio; SE=standard error

**Figure S1. Prevalence of acute seizures in preschool children by age group**


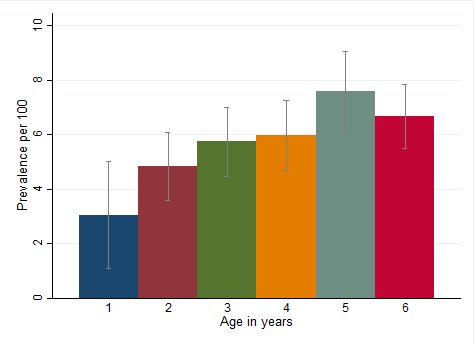


**Figure S2. The overlap of focal, repetitive and prolonged phenotypes of acute seizures in 221 preschool children**

Repetitive seizures

Prolonged

seizures

32 (15%)

51 (23%)

58 (26%)

19 (9%)

19 (9%)

28 (13%)

13 (6%)

Focal Seizures
